# A pH-Responsive Polyetheretherketone Implant Modified with a Core–Shell Metal–Organic Framework to Promote Antibacterial and Osseointegration Abilities

**DOI:** 10.34133/bmr.0188

**Published:** 2025-04-25

**Authors:** Shiqing Ma, Shiyu Yao, Yumeng Li, Yilin Yang, Tianyi Tong, Hong Zheng, Beibei Ma, Pengfei Wei, Zhengyi Di, Bo Zhao, Jiayin Deng

**Affiliations:** ^1^ Department of Stomatology, The Second Hospital of Tianjin Medical University, Tianjin 300070, PR China.; ^2^ Tianjin Key Laboratory of Oral Soft and Hard Tissues Restoration and Regeneration, Tianjin 300070, PR China.; ^3^ Department of Periodontology, Tianjin Medical University School and Hospital of Stomatology, Tianjin 300070, PR China.; ^4^ Beijing Biosis Healing Biological Technology Co., Ltd, Beijing 102600, PR China.; ^5^ College of Chemistry, Tianjin Key Laboratory of Structure and Performance for Functional Molecules, Tianjin Normal University, Tianjin 300387, PR China.

## Abstract

Polyetheretherketone (PEEK) is considered to be a potential material for oral implants due to its elastic modulus being similar to that of human cortical bone. However, the poor antibacterial, anti-inflammatory, and osseointegration properties of bioinert PEEK have hindered its clinical application. Therefore, this study designed and constructed a pH-responsive PEEK implant with a bilayer core–shell zeolitic imidazolate framework-8 (ZIF-8) structure loaded on its surface, with an antimicrobial peptide (KR12) encapsulated in the outer shell and an osteogenic peptide (osteogenic growth peptide ) encapsulated in its inner core. In this study, the bilayer core–shell ZIF-8 structure was confirmed to have pH-responsive properties. In vitro studies proved that the implant could promote bone marrow mesenchymal stem cells’ proliferation and differentiation and the M1 phenotype to M2 phenotype conversion of RAW 264.7 and could inhibit bacterial adhesion and proliferation. By constructing rats’ distal femur with/without infection models, it was further demonstrated that the novel implant could effectively inhibit bacterial adhesion and growth, inhibit inflammation, and promote peri-implant osseointegration, which was more substantial when the local area was infected and the pH was lower than that of normal tissue. Collectively, the results suggest that this novel pH-responsive PEEK implant loaded with a bilayer core–shell ZIF-8 structure is a promising peptide delivery implant system, which is well suited for dental applications and offers a potential solution for the prevention of infection during the early phase after implantation.

## Introduction

Artificial implants have gradually become one of the main treatments for edentulous and dentition defect patients. Implant osseointegration was described by Branemark [1] as “a direct structural and functional connection between bone and the load-carrying implant surface”. The main clinical manifestation of poor osseointegration is implant loosening. Stress shielding is the main cause of implant aseptic loosening during long-term service [2]. This is due to the insufficient load transfer between the bone and the implant. Based on Wolff’s law, bone growth is positively correlated with loading. Bone resorption occurs in the corresponding areas when the bone is left unloaded for long. Since the implant with a greater elastic modulus transmits most of the load, only part of the load is transferred to the bone. It would lead to bone loss at the bone–implant interface, resulting in inadequate osseointegration and ultimately implant loosening [3]. Conventional metal implants, such as titanium and titanium alloys, and nonmetallic materials, such as zirconia, are currently the most common materials for dental implants. However, their inherent high elastic modulus leads to stress-shielding effects in long-term applications, limiting their further development [4]. More recent research focused on implants made of polymer compounds, such as polyetheretherketone (PEEK) to overcome these limitations.

PEEK has become the favored polymer compound material in orthopedic/dental implants because of its excellent mechanical properties and superior biocompatibility [5]. The elastic modulus of PEEK is approximately equal to that of human cortical bone [6]. Consequently, PEEK can effectively eliminate stress shielding and prevent implant loss due to bone lesions and resorption caused by stress shielding. Additionally, PEEK has radiolucency, magnetic resonance imaging compatibility, fatigue resistance, and excellent chemical resistance [7]. The US Food and Drug Administration has recently certified medical-grade PEEK as the best long-term implant material, and it is widely applied in orthopedics, trauma treatments, spinal implants, and joint replacement [8]. Despite these advantages, regrettably, the bioinertness and inferior osteoconduction of PEEK have become a hindrance to its clinical application [9]. Herewith, the surface modification of PEEK is essential to promote osseointegration for its clinical application. In general, sulfonation could give PEEK a rough and porous surface and could provide a target for other surface modification methods through sulfonic acid groups. It has been widely used in the pretreatment of various PEEK surface modification methods [10].

Recently, bioactive peptides have received increasing attention due to their inherent low immunogenicity, biodegradability, bioactivity, and easy synthesis. With the development of bioscience and technology, the controllable spatial structure and loadable properties of peptides hold promise for broader applications in PEEK surface modification. Osteogenic growth peptide (OGP) is a naturally occurring 14-mer peptide that possesses the ability to enhance proliferation and osteogenic differentiation in osteoblast cell lines, including mesenchymal stem cells (MSCs) [11], and is reported to accelerate the implant osseointegration process efficiently [12]. It might possess the potential to modify the osteogenic properties of PEEK surfaces. Bacteria invade and adhere to the surface of the implant through the unhealed mucosa incision at the initial stage of implantation, whereafter it will cause peri-implant mucositis and peri-implantitis, which leads to early implant failure [13]. Therefore, it is crucial to modify the antibacterial properties of PEEK surfaces to control the initial adhesion and growth of bacteria on the implant surface. Antimicrobial peptides (AMPs) have broad-spectrum and potent antibacterial capabilities against drug-resistant bacteria. AMPs can disrupt bacterial cell membranes and induce their cleavage [14]. LL-37 is the only natural human cathelicidin in the AMP subfamily [15]. KR12 is the shortest derivative peptide retaining antibacterial activity and the core amphiphilic helical structure of LL-37, which has the advantages of low synthetic cost and low cytotoxicity [16]. Hence, in this study, OGP and KR12 were introduced to modify the surface of PEEK, expecting PEEK to have both antibacterial and osteogenic abilities.

Similar to that of the tumor microenvironment, the pH of the infected site microenvironment is lower than that of normal tissue [17]. Encouraged by the successful application of pH-responsive materials to tumor therapy [18], the acidic infected microenvironment may also serve as an “endogenous switch” for pH-responsive drug delivery for the prevention and treatment of infections in the early stages of implantation. Zeolitic imidazolate framework-8 (ZIF-8) is a tetrahedral structural unit formed by bonding Zn^2+^ ions and 2-methylimidazole linkers at specific angles and orientations. ZIF-8 combines a highly microporous structure (specific surface area of over 1,000 m^2^/g) with the stability of inorganic zeolites, attracting great attention [19,20]. It also has other outstanding features, such as a regular pore structure, high porosity, biodegradability, and low cytotoxicity, which are ideal for drug delivery. Zinc on ZIF-8 can induce macrophage polarization to an anti-inflammatory phenotype (M2), exhibiting immunomodulatory effects [21]. Interestingly, the framework structure of ZIF-8 is relatively stable in normal environments, whereas it will accelerate degradation under the inflammatory environment’s acidic conditions (pH < 7) and release the metal ions and drug molecules encapsulated therein [22]. The above properties make it an ideal pH-responsive drug delivery system. Therefore, the loading of ZIF-8 onto the PEEK surfaces and encapsulating 2 peptides within it has the potential to construct pH-responsive implants that could cope with different conditions in vivo.

**Fig. 1.**
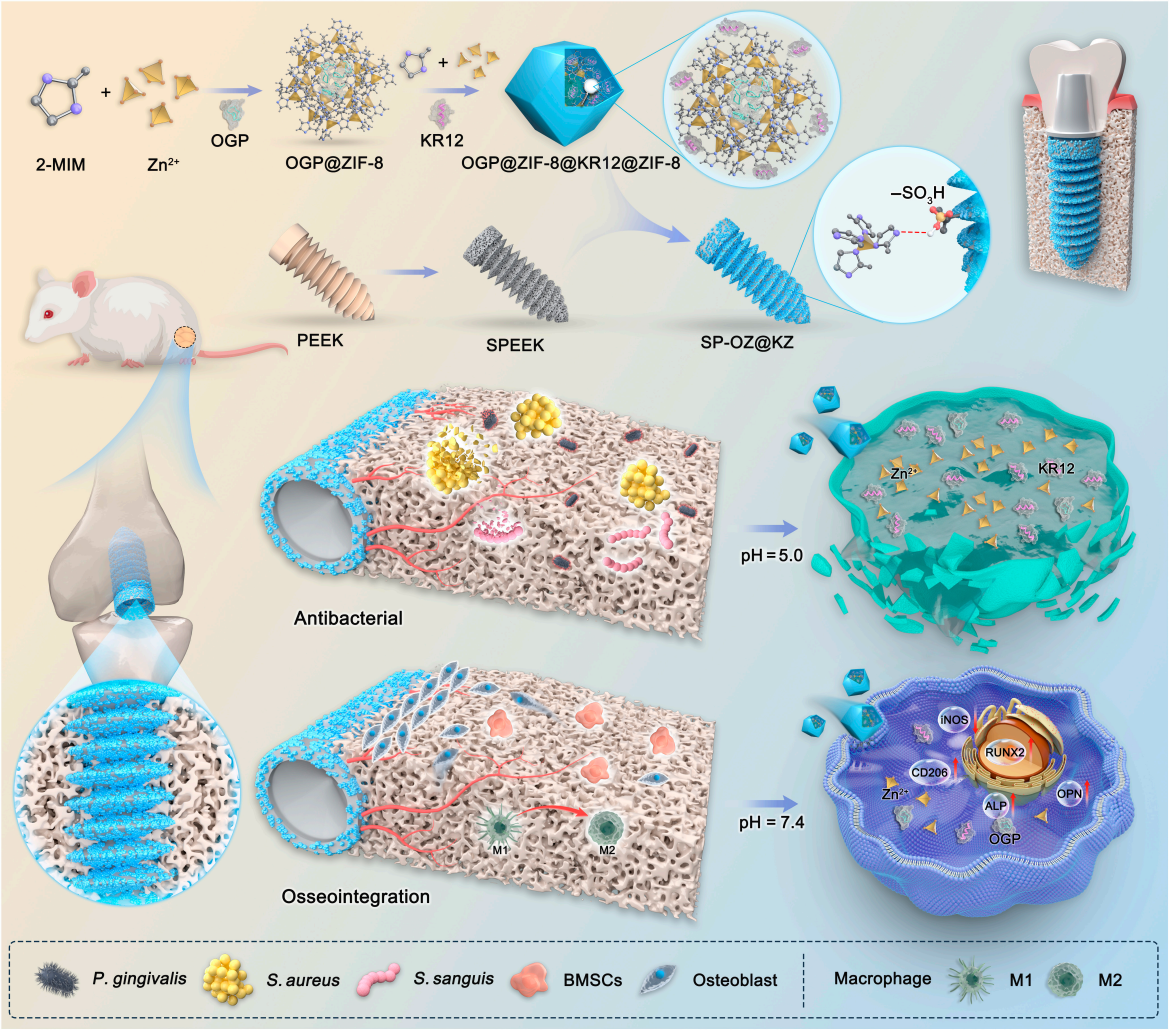
The schematic of this study. ZIF-8, zeolitic imidazolate framework-8; OGP, osteogenic growth peptide; 2-MIM, 2-methylimidazole; PEEK, polyetheretherketone; SPEEK, sulfonated PEEK; SP-OZ@KZ, SPEEK-OGP@ZIF-8@KR12@ZIF-8; iNOS, inducible nitric oxide synthase; RUNX2, runt-related transcription factor 2; ALP, alkaline phosphatase; OPN, osteopontin; BMSCs, bone marrow mesenchymal stem cells.

The process of peri-implant osteogenesis involves a reduction in primary stability and the development of secondary stability. It is well known that the rate of primary stability loss is faster than the development of secondary stability. As a result, a sudden decrease in stability would occur within 1 to 8 weeks after implant surgery [23]. Therefore, the release of components on the implant surface that promote osseointegration should be delayed and prolonged as much as possible. Based on the above properties, we comprehensively designed and constructed a pH-responsive bilayer ZIF-8 core–shell structure with OGP wrapped in the inner core and KR12 wrapped in the outer shell, which was encapsulated on the surface of sulfonated PEEK (SPEEK) (SPEEK-OGP@ZIF-8@KR12@ZIF-8). We speculated that this multifunctional coated implant could provide significant advantages in different situations (Fig. 1): (a) In a normal physiological environment, the core–shell ZIF-8 structure slowly degraded and released small amounts of Zn^2+^ and KR12 to cope with bacterial adhesion at the initial stage of implantation while promoting localized osseointegration. Over time, the OGP encapsulated in the inner core gradually released and contributed to the increase of secondary stability. (b) In an inflammatory environment, the core–shell ZIF-8 structure rapidly degraded and released large amounts of Zn^2+^ and KR12 for sterilization, while Zn^2+^ converted macrophages to an M2-phase anti-inflammatory phenotype in response to the occurred infection and released OGP at the same time to assist the promotion of rapid localized osseointegration.

In this study, we proposed to construct pH-responsive bilayer ZIF-8 wrapped peptide-modified PEEK implants through a series of material characterization as well as in vivo and in vitro experiments to verify their osteogenetic, anti-inflammatory, and antibacterial properties, so as to provide a therapeutic strategy for the prevention of peri-implantitis and offer a promising clinical application in the field of implantology.

## Materials and Methods

### Materials

Biomedical-grade PEEK substrates were cut into different dimensions. Disk (10-mm diameter and 1-mm thickness) samples were used for in vitro studies, while rods (2-mm diameter and 6-mm length) samples were used for in vivo studies. Acetone and sulfuric acid (H_2_SO_4_, 95% to 98%) were purchased from Chuandong Chemical Company (Chongqing, China). 2-Methylimidazole (99%), zinc nitrate (Zn(NO_3_)_2_·6H_2_O, 99%), methanol, and ethanol were purchased from Aladdin Industrial Co. (Shanghai, China). OGP (ALKRQGRTLYGFGG, >90%) and KR12 (KRIVQRIKDFLR, >90%) were purchased from Curtius Biotech (China).

### Sample preparation

#### Pretreatment of PEEK substrates

PEEK substrates were polished and rinsed sequentially with acetone, ethanol, and double-distilled H_2_O for 10 min each at room temperature. The substrates were then dried at 60 °C in a vacuum oven for 4 h. Afterward, the PEEK substrates were treated with concentrated sulfuric acid with ultrasonic agitation at room temperature for 3 min. The SPEEK substrates were then washed in acetone to remove the residual sulfuric acid. The SPEEK substrates were washed in deionized water 3 times (10 min per time) and treated by hydrothermal treatment at 100 °C for 1 h to remove the residues, subsequently. Experimental SPEEK samples were obtained after drying in a vacuum oven at 60 °C.

#### Synthesis of SPEEK-ZIF-8, SPEEK-OGP@ZIF-8, SPEEK-KR12@ZIF-8, and SPEEK-OGP@ZIF-8@KR12@ZIF-8

Four groups of ZIF-8 particles were prepared using a slightly modified precipitation method based on previous studies [24]. Briefly, after Zn(NO_3_)_2_·6H_2_O was completely dissolved in methanol, a 2-methylimidazole methanol solution was gradually added into the Zn(NO_3_)_2_ solution. The mixture was stirred for 1 h at room temperature. ZIF-8 was collected by centrifugation (10,000 rpm, 10 min), washed 3 times with methanol, and dried under vacuum. OGP@ZIF-8 and KR12@ZIF-8 were fabricated via a similar method. OGP and KR12 were dissolved in the 2-methylimidazole methanol solution, respectively. The solutions were then added to the Zn(NO_3_)_2_·6H_2_O methanol solution, respectively. The mixtures were stirred for 1 h at room temperature, and OGP@ZIF-8 and KR12@ZIF-8 were collected by centrifugation, then washed 3 times with methanol, and dried under vacuum. The ZIF-8 shell grew spontaneously onto the OGP@ZIF-8 core owing to isotropy. KR12 was wrapped into the ZIF-8 shell by a one-pot process at the same time. For subsequent coating, the SPEEK samples were immersed in ZIF-8, OGP@ZIF-8, KR12@ZIF-8, and OGP@ZIF-8@KR12@ZIF-8 solution (1 mg/ml in 10 mM Tris-HCl buffer, pH = 8.5), respectively, and incubated overnight at room temperature. The samples were then rinsed with deionized water and ultrasonicated for 20 s to remove any unabsorbed product or reactant. After drying under vacuum, SPEEK-ZIF-8 (SP-Z), SPEEK-OGP@ZIF-8 (SP-OZ), SPEEK-KR12@ZIF-8 (SP-KZ), and SPEEK-OGP@ZIF-8@KR12@ZIF-8 (SP-OZ@KZ) were then obtained.

#### Peptide loading efficiency

OGP@ZIF-8, KR12@ZIF-8, and OGP@ZIF-8@KR12@ZIF-8 nanoparticles (NPs) for detection of the peptide loading rate and peptide release rate were prepared using fluorescently labeled OGP and KR12 (rhodamine B-OGP and fluorescein isothiocyanate [FITC]-KR12).

The supernatants after rhodamine B-OGP@ZIF-8, FITC-KR12@ZIF-8, and rhodamine B-OGP@ZIF-8@FITC-KR12@ZIF-8 fabrication were collected by centrifugation. A fluorescence spectrometer (PerkinElmer, USA) was used to measure the fluorescence intensity of the supernatants, respectively. The fluorescence value was converted into peptide content by a standard curve (Fig. S1). Peptide loading efficiency (LE) was calculated using the following formula:

$$\;\text{LE}\;\left(\%\right)=1-\frac{W_{0}}{W_{1}}*100\%$$ (1)

*W*_0_ represents the peptide content in the supernatant, and *W*_1_ represents the peptide dosage during the NPs’ fabrication process. The experiment was repeated 3 times (*n* = 3), and the mean value was obtained.

### Sample characterization

The size distributions of the NPs were evaluated by dynamic light scattering (DLS; Zetasizer Nano S90, Malvern, UK). The morphology and shape of NPs and the surface morphologies of the coated SPEEK samples were observed by scanning electron microscopy (SEM, Mira4, Tescan, Czech Republic; Gemini 300, Zeiss, Germany) and transmission electron microscopy (TEM, Talos F200S, FEI, USA). The structure and the elemental distribution of the NPs were also analyzed by TEM (Talos F200S, FEI, USA). The crystal structures of the NPs were detected by powder x-ray diffraction (XRD; D8 ADVANCE, Bruker, Germany). The functional groups of the NPs were determined by attenuated total reflectance−Fourier transform infrared spectroscopy (Nicolet iS50, Thermo Fisher Scientific, USA). The surface hydrophilicity of the samples was evaluated by contact angle measurements (JGW-360A, China). A 3M commercial tape with an adhesion force greater than 1.23 N/cm was used to bond to the sample surface and was torn off after applying a force of 1 kg to the surface. The shedding of NPs on the surface was observed by SEM (Mira4, Tescan, Czech Republic; Gemini 300, Zeiss, Germany) and quantified by the ImageJ software. The tensile strength of the samples was tested by using an Instron 3367 universal testing machine tester at room temperature, with a cross-head speed of 1 mm/min.

### pH-responsive peptide release behavior

To characterize the pH degradability of the NP coatings, the samples were soaked in phosphate-buffered saline (PBS) buffer with pH = 7.4 and 5.0, respectively. The amount of Zn^2+^ released from the NP coatings was evaluated by inductively coupled plasma mass spectrometry (Agilent, 7700 series, USA). The amount of fluorescence-labeled peptides released from the NP coatings was evaluated by a fluorescence spectrometer (PerkinElmer, USA). The morphology of the NP coatings after being soaked in PBS buffer with pH = 7.4 and 5.0 for 12 and 72 h was observed by SEM.

### Antibacterial capacity in vitro

#### Bacterial culture and morphology

The 6 group samples (PEEK, SPEEK, SP-Z, SP-OZ, SP-KZ, and SP-OZ@KZ) were sterilized in alcohol overnight and placed in a 24-well plate. Then, the samples were incubated with 1 ml of bacterial suspension containing 1 × 10^5^ CFU/ml *Staphylococcus aureus*, *Streptococcus sanguis*, and *Porphyromonas gingivalis* at different pH values (pH = 7.4 and 5.0) separately. After incubation at 37 °C for 24 h, the bacteria were immobilized with glutaraldehyde solution and dehydrated gradually with gradient ethanol (30, 50, 75, 85, 95, and 100% v/v), respectively, for 10 min each, dried under the vacuum critical point, and sputter coated with gold. The bacteria on the surface of the samples were observed by SEM.

#### Antibacterial rate

To evaluate the antibacterial rate, samples were incubated with 1 ml of bacterial suspension containing 1 × 10^5^ CFU/ml *S. aureus*, *S. sanguis*, and *P. gingivalis* in Luria–Bertani media with different pH values at 37 °C separately. The samples were taken out and rinsed 3 times with PBS to remove nonadherent bacteria. Then, the adherent bacteria on the surface were eluted and collected via ultrasonic treatment. The eluent was diluted 100 times to make a bacterial suspension, and 30 μl of the suspension was seeded onto sterile agar plates and incubated for 24 h. The antibacterial rate was calculated using the following formula:

$$\text{Antibacterial rate}\;\left(\%\right)=\frac{\left(A-B\right)}{A}*100\%$$ (2)

*A* represents the average number of bacterial colonies in the control group (PEEK samples, CFU/ml), while *B* represents the average number of bacterial colonies in the experimental groups (CFU/ml).

#### Bacteriostatic ring

The antibacterial capacities of the samples were determined by the bacteriostatic ring test according to the National Standard of China GB/T 2738-2012 protocol. Briefly, 30 μl of bacterial suspension containing 1 × 10^8^ CFU/ml *S. aureus*, *S. sanguis*, and *P. gingivalis* was separately spread onto plate media with different pH values. The samples were then gently placed on the center of the agar surface. After culturing at 37 °C, the antibacterial capacity was assessed based on the width of the inhibition zone around the samples.

#### Live/dead staining

After incubation with *S. aureus*, *S. sanguis*, and *P. gingivalis* suspension in Luria–Bertani media with different pH values at 37 °C separately, the samples were rinsed to remove the nonadherent bacteria, and the adherent bacteria on the surface were stained with a live/dead staining kit (Life Technologies Corporation, CA). The dead bacteria (red) and live bacteria (green) were observed with a fluorescence microscope (Olympus, Tokyo, Japan).

### In vitro cytotoxicity

#### Cells culture

Mouse macrophage cells (RAW 264.7, Procell, China) and rat bone marrow mesenchymal stem cells (rBMSCs, Procell, China) were used in the in vitro study. The cells were cultured in Dulbecco’s modified Eagle’s medium supplemented with 1% penicillin–streptomycin (Solarbio, China) and 10% fetal bovine serum (TBD Science, China) at 37 °C with 5% CO_2_. At approximately 80% confluence, the cells were passaged by 0.05% trypsinization (Solarbio, China), and the cells of the third to fifth passages were utilized in the in vitro studies.

#### Cytocompatibility evaluation

The samples were placed in a 24-well plate, and then 1 × 10^4^ cells were seeded onto each sample and incubated at 37 °C for 1, 3, 5, and 7 d. Then, the metabolites produced by cells on the samples were detected by Cell Counting Kit-8 (CCK-8; New Cell & Molecular, China) to evaluate the cytocompatibility of the samples. The untreated cell group was included in this experiment as a control group. The absorbance was determined at 450 nm by a microplate reader (Molecular Devices, SpectraMax Plus 384, USA).

#### Live/dead staining assay

The cytotoxicity of the samples was determined by live/dead staining. Cells (5 × 10^4^) were seeded onto the samples and incubated for 1 and 3 d. At a specified time point, the samples were washed with PBS 3 times and stained with an acridine orange/ethidium bromide staining kit (Sangon Biotech, China). Then, the samples were washed with PBS 3 times again and observed with a fluorescence microscope (Olympus, Tokyo, Japan). The living cells were green, while the dead cells were red under the microscope.

### In vitro osteogenic activity

#### ALP staining

The rBMSCs (1 × 10^4^) were calculated with the leach liquor of each sample in 24-well plates. When the cell density rose to 80%, the growth medium was replaced with the osteogenic induction medium, which contained 10% fetal bovine serum, 1% vitamin C, and 0.2% dexamethasone. The alkaline phosphatase (ALP) activity of rBMSCs was measured after being cultured with osteogenic induction medium for 7, 14, and 21 d, respectively. Then, the cells were stained by a BCIP/NBT (5-bromo-4-chloro-3-indolyl phosphate/nitrotetrazolium blue chloride) ALP color development kit (Beyotime, China) to characterize the intracellular ALP qualitatively. The photographs were recorded with an optical microscope (Olympus, Tokyo, Japan).

#### Alizarin red staining

Alizarin red staining (ARS) was used to evaluate the mineralization of the extracellular matrix (ECM) of rBMSCs; 1 × 10^4^ rBMSCs were incubated with the leach liquor of each sample in osteogenic induction medium for 7, 14, and 21 d and fixed with 4% paraformaldehyde. After staining with alizarin red, the excess dyes were washed with PBS and then the cells were observed and photographed.

#### Immunofluorescence staining

After being incubated with the leach liquor of each sample in an osteogenic induction medium for 3 d, the bone marrow mesenchymal stem cells (BMSCs) were fixed with 4% paraformaldehyde and permeabilized with Triton X-100 (Sigma, USA) for 5 min, washed with PBS 3 times, and blocked for 30 min. Cells were incubated with the following primary antibodies: anti-ALP (1:200, Abcam, UK) and anti-runt-related transcription factor 2 (anti-RUNX2) (1:200, Abcam, UK) overnight at 4 °C. Cells were then incubated with Cyanine3-conjugated anti-rabbit secondary antibody (1:1,000, Abcam, UK) under darkness for 1 h. The nuclei were stained with 4′,6-diamidino-2-phenylindole (DAPI) solution, subsequently. The cells were observed by a confocal laser scanning microscope (LSM 800, Zeiss, Germany).

#### Osteogenic gene expression

The messenger RNA (mRNA) expression of the genes related to osteogenesis, such as *ALP*, *Runx2*, and osteopontin (*OPN*), was analyzed by the real-time quantitative reverse transcription polymerase chain reaction (RT-qPCR). The total mRNA was extracted by a total RNA kit (Yeasen, China) after culturing in an osteogenic medium for 7 and 14 d and converted to complementary DNA by a reverse transcription kit (Yeasen, China). Then, SYBR Green Master Mix (Yeasen, China), primers, and complementary DNA were mixed in a 96-well PCR plate and PCR was performed through a Roche LC480II system (Roche, Switzerland). The primer sequences were designed (Sangon Biotech, China) and are shown in Table S1. All expression levels were normalized with glyceraldehyde-3-phosphate dehydrogenase (GAPDH) and quantified using the comparison 2^−ΔΔCt^ method.

#### Osteogenic protein expression

Western blot was conducted to assess the protein expression levels of RUNX2, ALP, and OPN. After culturing for 7 and 14 d, the protein from rBMSCs was separated via sodium dodecyl sulfate–polyacrylamide gel electrophoresis and transferred to polyvinylidene fluoride (PVDF) membranes (Merck Millipore, Ireland). Then, the membranes were blocked in 5% skimmed milk for 1 h. The PVDF was coincubated with the primary antibodies anti-RUNX2 (1:5,000, Huabio, China), anti-ALP (1:5,000, Huabio, China), anti-OPN (1:1,000, Proteintech, China), and anti-GAPDH (1:5,000, Huabio, China) at 4 °C overnight and then coincubated with a corresponding horseradish peroxidase-conjugated secondary antibody (1:1,000, Beyotime, China) for 1 h at room temperature. An enhanced chemiluminescent Western blot analysis system (New Cell & Molecular, China) was used to detect antibody-bound proteins. The intensity of the proteins was quantified by the ImageJ software and normalized by the value of GAPDH.

### In vitro evaluation of macrophage polarization

#### Immunofluorescence staining

The polarization of macrophages was investigated by immunofluorescence staining of the surface markers of M1 macrophages (inducible nitric oxide synthase [iNOS]), M2 macrophages (CD206), interleukin 1β (IL-1β), and interleukin 10 (IL-10). After incubation with the leach liquor of each sample for 3 d, the RAW 264.7 cells were fixed, permeabilized, and blocked following the method in the “Immunofluorescence staining” section. Cells were incubated with anti-iNOS (1:200, Abcam, UK), anti-CD206 (1: 200, Proteintech, USA), anti-IL-1β (1:200, Abcam, UK), and anti-IL-10 (1:200, Abcam, UK) overnight at 4 °C. Cells were then incubated with secondary antibody. The nuclei were stained with DAPI solution, subsequently. The cells were observed by confocal laser scanning microscopy.

#### Inflammatory gene expression

The expression of inflammatory genes was analyzed using RT-qPCR (QuantStudio 1, ABI, USA) following the method in the “Osteogenic gene expression” section to examine *IL-1β*, *iNOS*, *CD206*, and *IL-10* expression.

#### Cytokine assessment by ELISA kits

Levels of pro-inflammatory (IL-1β) and anti-inflammatory (IL-10) cytokines in cell culture supernatants were measured by enzyme-linked immunosorbent assay (ELISA) kits (BioLegend) according to the manufacturer’s instructions.

#### Macrophage surface marker protein expression

Western blot was conducted to assess the protein expression level of iNOS and CD206. After culturing for 3 d, the protein from RAW 264.7 cells was separated via sodium dodecyl sulfate–polyacrylamide gel electrophoresis and transferred to PVDF membranes (Merck Millipore, Ireland). Then, the membranes were blocked in 5% skimmed milk for 1 h. The PVDF was coincubated with the primary antibodies anti-iNOS (1:1,000, Huabio, China) and anti-CD206 (1:1,000, Huabio, China) at 4 °C overnight and then coincubated with a corresponding horseradish peroxidase-conjugated secondary antibody (1:1,000, Beyotime, China) for 1 h at room temperature. The enhanced chemiluminescent Western blot analysis system (New Cell & Molecular, China) was used to detect antibody-bound proteins. The intensity of the proteins was quantified by the ImageJ software and normalized by the value of GAPDH.

### In vivo animal experiment

#### Surgical implantation

Animal experiments strictly complied with the regulations of the Tianjin Medical Experimental Animal Care. All protocols were approved by the Institutional Animal Care and Use Committee of Yi Shengyuan Gene Technology (Tianjin, China) (protocol number YSY-DWLL-2023342).

Eight-week-old male Sprague Dawley rats were randomly separated into 6 groups (PEEK group, SPEEK group, SP-Z group, SP-OZ group, SP-KZ group, and SP-OZ@KZ group; *n* = 12). The rats were anesthetized with an intraperitoneal injection of 1% pentobarbital sodium, and then one leg was immobilized, shaved, and thoroughly sterilized with povidone iodine. The medial patellar skin was cut, and the femoral condyle was exposed. A 2-mm-diameter hole was then drilled through the femoral condyle along the long axis of the femur. Each group’s implant (Ф 2 mm × 6 mm) was then placed, and the incision was carefully sutured. After implantation for 1, 4, and 12 weeks, 3 rats from each group were sacrificed, and the femur was removed with the implant and fixed with formalin for further experiments.

The femoral condyle implantation area infection model was fabricated similarly to the method described above; 10 μl of *S. aureus* suspension (1 × 10^5^ CFU/ml) was injected into the drilling site before the implants were placed in operation areas.

#### Micro-computed tomography analysis

Micro-computed tomography (micro-CT; SkyScan 1276, Germany) was used to analyze the new bone formation around the implants after implantation for 1, 4, and 12 weeks. Three-dimensional (3D) images were reconstructed by CTVox, and the bone volume over total volume, trabecular thickness, trabecular number, and trabecular separation were calculated by CTAn.

#### Histological analysis

Histological and immunohistochemistry (IHC) staining was performed after implantation for 1, 4 and 12 weeks. For histological analysis, the fixed femur was decalcified in EDTA solution (Solarbio, China) and dehydrated by using a graded series of alcohol. After being embedded in paraffin, the samples were cut into 5-μm-thick sections and stained with hematoxylin–eosin (HE) and Masson’s trichrome stains (Baiqiandu, China). The sections were observed by an optical microscope (Eclipse E100, Nikon, Japan) and photographed by an imaging system (DS-U3, Nikon, Japan).

#### IHC staining analysis of osteogenesis

IHC staining was conducted to detect the expression levels of collagen-1 (COL-1) and osteocalcin (OCN) to assess the osteogenesis situation. Briefly, anti-COL-1 (1:500, ab8448, Abcam, UK) and anti-OCN (1:500, ab13420, Abcam, UK) primary antibodies were incubated with the sections derived after 4 and 12 weeks of in vivo implantation overnight at 4 °C. After that, the sections were incubated with the corresponding secondary antibodies. Finally, the sections were developed with 3,3′-diaminobenzidine tetrahydrochloride (DAB) and counterstained with hematoxylin. The sections were then observed and photographed.

#### Inflammation and microbiological evaluation

After infection model fabrication for 1 week, the granulation tissue around the surgical area was collected and immersed in physiological saline for 2 h at room temperature; 30 μl of the supernatant of the solution was transferred to aseptic agar plates and incubated at 37 °C for 24 h to observe colony formation.

Fixed femur samples were obtained after implantation for 1 week, and the samples were cut into 5-μm-thick sections and stained with tartrate-resistant acid phosphatase (TRAP; Nanjing Jiancheng, China).

The sections of femur samples were incubated with anti-iNOS (1:500, ab8448, Abcam, UK) and anti-CD206 (1:500, ab8448, Abcam, UK) primary antibodies overnight at 4 °C. After that, the sections were incubated with the corresponding secondary antibodies. Finally, the sections were developed with DAB and counterstained with hematoxylin.

### Statistical analysis

SPSS 21.0 (IBM, USA) was used for statistical analysis. A Student *t* test was used to analyze parametric data, and one-way analysis of variance (ANOVA) was used for multiple group comparisons. All quantitative data are presented as mean ± standard deviation. When *P* < 0.05 (*) and *P* < 0.01 (**), the difference was considered statistically significant.

## Results and Discussion

### Synthesis and characterization of SP-OZ@KZ

To modify PEEK to possess antimicrobial and osteogenic properties, this study designed the encapsulation of OGP and KR12 peptides in ZIF-8 and loaded the bilayer core–shell ZIF-8 on the SPEEK surface. For the synthesis of each group of ZIF-8 NPs loaded onto SPEEK in this study, the OGP and KR12 peptide solutions were separately mixed with a 2-methylimidazole solution first; afterward, they were mixed with the Zn(NO_3_)_2_ solution to induce the self-assembly and biomineralization of ZIF-8. The LE of the OGPs was measured as 83.3%, while that of the KR12 peptide in the KZ groups was 86.1%. When the KR12 peptide was encapsulated in the shell layer, the efficiency decreased to 82.2%. As suspected, the relatively high peptide LE of ZIF-8 is related to its unique framework structure.

**Fig. 2.**
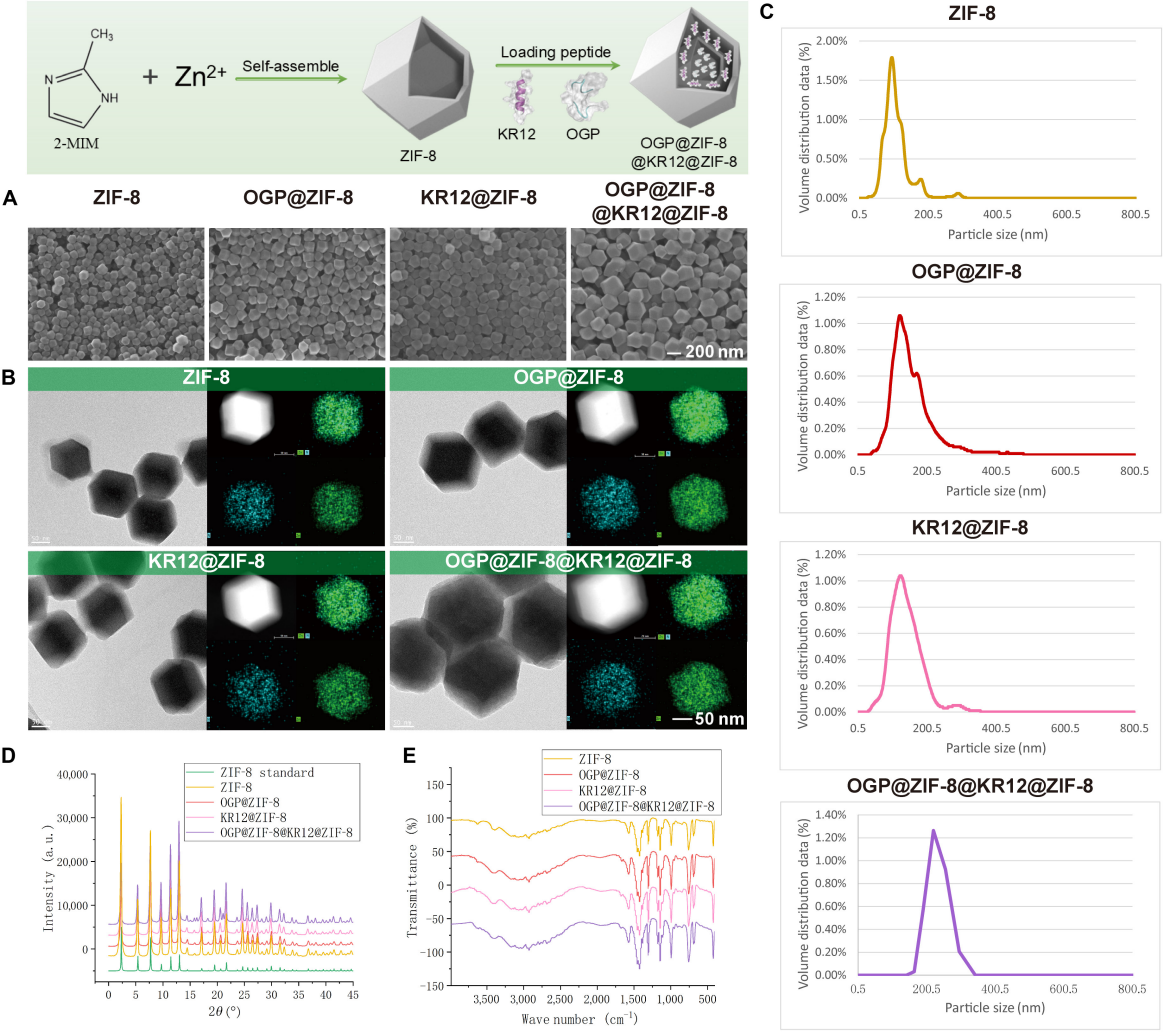
Chemical and physical properties of nanoparticles. (A and B) The morphology and elemental mapping of nanoparticles visualized by scanning electron microscopy (SEM) and transmission electron microscopy (TEM). (C) Particle size distribution of nanoparticles. (D) X-ray diffraction (XRD) spectrum of nanoparticles. (E) Fourier transform infrared (FTIR) spectrum of nanoparticles. 2-MIM, 2-methylimidazole.

The morphology of ZIF-8, OGP@ZIF-8, KR12@ZIF-8, and OGP@ZIF-8@KR12@ZIF-8 was observed by SEM and TEM (Fig. 2A and B), which revealed that the ZIF-8 fabricated in this study exhibited a uniform, rhombic dodecahedral morphology. The peptide-loaded NPs still exhibited the same uniform dodecahedral structure as ZIF-8, indicating that OGP and KR12 loading did not disrupt the microstructure of ZIF-8. The DLS measurement showed that the average sizes of OGP@ZIF-8 and KR12@ZIF-8 were 142.4 ± 1.2 and 134 ± 4.7 nm, respectively, which were larger than that of ZIF-8 (99.7 ± 2.6 nm) and smaller than that of OGP@ZIF-8@KR12@ZIF-8 (228 ± 3.58 nm) (Fig. 2C). The results of DLS were consistent with the particle sizes observed in SEM and TEM, indicating that bilayer ZIF-8 was successfully fabricated and the peptides were successfully encapsulated in the monolayer and bilayer ZIF-8 framework structure. Analysis of the patterns of 4 groups of NPs by powder XRD indicated the appearance of the main distinct peaks of ZIF-8, which reconfirmed the successful synthesis of ZIF-8 and that the encapsulation of the peptide did not affect the crystalline structure of ZIF-8 (Fig. 2D). However, the characteristic diffraction peaks of peptides were not evident in the XRD spectra of OGP@ZIF-8, KR12@ZIF-8, and OGP@ZIF-8@KR12@ZIF-8, which should be attributed to the encapsulation of the peptide in the framework structure of ZIF-8. The TEM elemental mapping results (Fig. 2B) verify the uniformly dispersed zinc in the 4 groups of NPs, while nitrogen was more concentrated in the center of OGP@ZIF-8, KR12@ZIF-8, and OGP@ZIF-8@KR12@ZIF-8, which further confirmed that the peptides were encapsulated in the ZIF-8 framework structure. The Fourier transform infrared spectra of the NPs are shown in Fig. 2E. All of the 4 groups of NPs showed the characteristic peaks of ZIF-8 according to the previous reference at about 3,400, 3,100, 3,000, 1,570, 1,457, 1,423, 1,382, 1,308, 1,146, 994, 759, 693, and 423 cm^−1^ [25]. The spectra of OGP@ZIF-8, KR12@ZIF-8, and OGP@ZIF-8@KR12@ZIF-8 showed that all of them contained weak C=O absorption bands representing peptide bonds at about 1,740 cm^−1^ [26]. That might be due to the peptide being encapsulated in the framework structure of ZIF-8, resulting in a shielding effect.

**Fig. 3.**
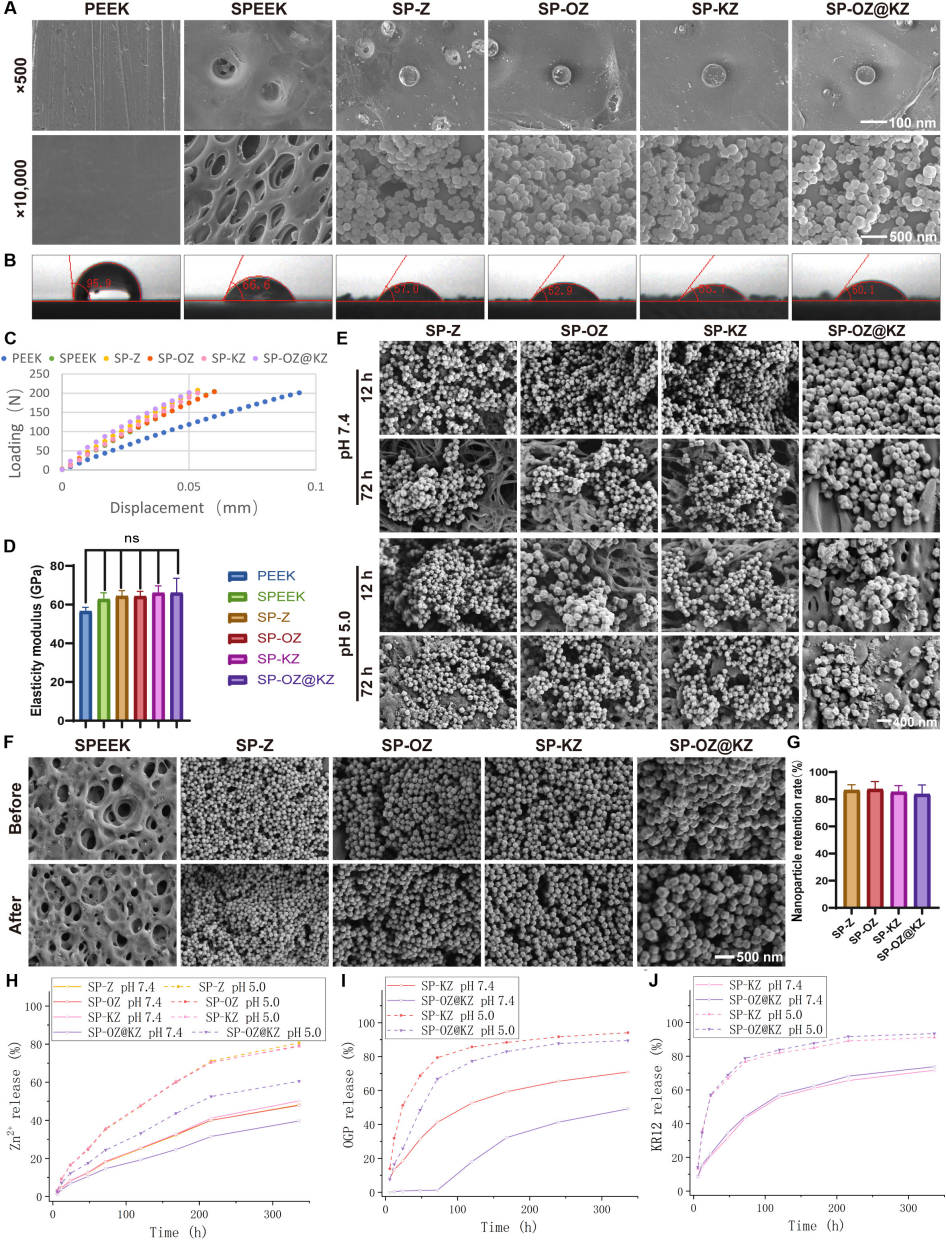
Chemical and physical properties of the PEEK samples. (A) The morphology of the PEEK samples at different magnifications visualized by SEM. (B) Water contact angle measurements of different samples. (C) The loading–displacement curves of each sample. (D) The elasticity modulus of each sample. (E) SEM images of nanoparticle (NP) coating after immersion at pH 5.0 and 7.4 for 12 and 72 h. (F and G) The morphology before and after tape tearing and NP retention rate of each sample. (H to J) Zn^2+^, OGP, and KR12 peptide release of the PEEK samples. SP-Z, SPEEK-ZIF-8; SP-OZ, SPEEK-OGP@ZIF-8; SP-KZ, SPEEK-KR12@ZIF-8.

The surface morphology of the 6 groups of PEEK samples was observed by SEM in Fig. 3A. Compared with the relatively smooth surface of the PEEK group, a 3D porous surface was formed in the SPEEK group after sulfonation treatment. In the SP-Z, SP-OZ, SP-KZ, and SP-OZ@KZ groups, the NPs were embedded in the pores of the 3D porous rough surface of SPEEK. The contact angle was measured to confirm the surface hydrophilicity properties of the 6 group samples. As depicted in Fig. 3B, the contact angle values of PEEK, SPEEK, SP-Z, SP-OZ, SP-KZ, and SP-OZ@KZ were 95.9°, 66.6°, 57°, 52.9°, 55.7°, and 50.1°, respectively. The surface hydrophilicity of SPEEK was increased significantly after sulfonation treatment compared to that of PEEK. The contact angles of NP-coated SPEEK groups were decreased relative to that of the SPEEK group, while there was no significant difference between the 4 NP-coated groups. The hydrophilicity of the implant material is one of the key factors affecting osseointegration capacity. Previous studies showed that modifying the hydrophilicity of the material could promote the adhesion and differentiation of bone progenitor cells and accelerate mineralization on the bone–implant interface [27]. After the tape was bonded to the surface of each sample and torn off, the surface of each sample was examined by SEM. Figure 3F and G show that there was no significant change on the surface of SPEEK, and the analysis revealed that the NP retention on the surface of the SP-Z, SP-OZ, SP-KZ, and SP-OZ@KZ groups was about 86.5%, 87.3%, 85.2%, and 83.6%, respectively. The stability of the surface coating of the implant is one of the bases for its expected function after implantation. Therefore, in this study, the bonding strength of the NPs loaded on the implant surface was initially tested by using 3M commercial tape bonding and then tearing off. The results showed that at least 80% of the NPs on the surface of the implants were retained, which was a relatively satisfactory retention rate. It has been reported that ZIF-8 and sulfonic acid groups can be combined through hydrogen bonding [28], which is an intermolecular force stronger than the van der Waals force. The binding of NPs to the SPEEK surface of each group had a certain degree of stability; this was attributed not only to hydrogen bonding but also to the mechanical embedding of the rough porous surface of SPEEK at the same time. The stability is expected to prevent implant failure caused by implant dysfunction by preventing NP detachment. The SPEEK, SP-Z, SP-OZ, SP-KZ, and SP-OZ@KZ groups showed a certain extent of changes in elasticity modulus versus the PEEK group (Fig. 3C and D). However, no significant differences were found between the groups.

The release behavior of the NP coating on the PEEK samples at different pH values was investigated to confirm the pH–response effect. Figure 3H to J show the time-dependent release profiles of Zn^2+^ and peptides in PBS solutions under different pH values. The release of Zn^2+^ and peptides was slow at pH 7.4. The OGP encapsulated in the inner layer of SP-OZ@KZ showed the most obvious difference in release at different pH values, which could be detected only in a trace amount during the first 3 d. Under acidic conditions, the coordination between Zn^2+^ and 2-methylimidazole will be disrupted, leading to the accelerated release of Zn^2+^ and peptides encapsulated in the ZIF-8 framework structure. At pH 5.0, after incubation for 5 d, OGP was released 85.69% ± 1.47% in SP-OZ, the KR12 was released 82.10% ± 3.07% and 83.80% ± 1.34% in SP-KZ and SP-OZ@KZ, respectively, while OGP was released 89.48% ± 1.80% in SP-OZ@KZ at 14 d. The release behavior at different pH values validated the concept of this study and confirmed the successful fabrication of the pH-responsive surface-coating-modified PEEK implant. To characterize the pH degradability of the NP coating on the PEEK samples, the morphology of the NP coating at different pH values was observed by SEM (Fig. 3E). The crystal could keep its morphology for 24 h at pH 7.4. However, the crystal structure rapidly disassembled in 24 h at pH 5.0. The complete collapse of the NPs could be observed after 12 h, reconfirming the pH sensitivity of the NP coating of the SPEEK.

### Antibacterial capacity in vitro

A bacteriostatic implant surface is essential, especially at the early implantation stage. Infection easily occurs due to the initial adhesion of bacteria onto the interfaces between the implant surface and unhealed surrounding soft tissue [29]. To achieve enduring success of implants, it is crucial to modify the implant surface with antibacterial properties to prevent bacterial adhesion. *P. gingivalis* was recognized as a pivotal pathogenic related to peri-implantitis [30]. Meanwhile, *S. sanguis* was confirmed to be a pioneering colonizer and plays a key role in aiding posterior bacterial adhesion and oral biofilm development [31]. Considering the broader clinical use of implants, the antibacterial effect of the samples against *S. aureus* was also detected in this study.

**Fig. 4.**
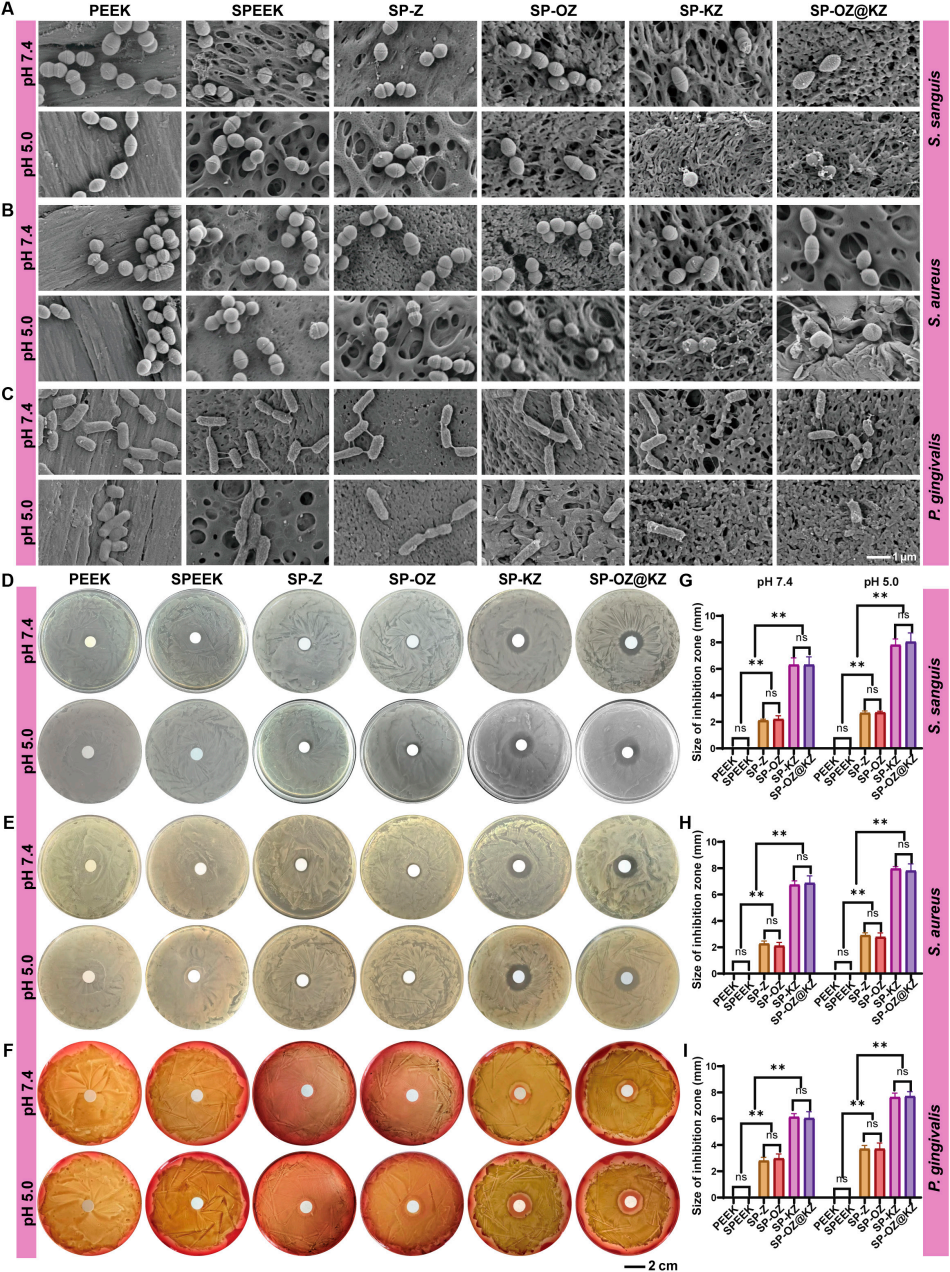
Evaluation of antibacterial properties of samples. (A to C) The quantity and morphology of the bacteria on the surfaces of the samples visualized by SEM. (D to F) Inhibition zones of *Staphylococcus aureus*, *Streptococcus sanguis*, and *Porphyromonas gingivalis* co-cultured with samples for 24 h. (G to I) Statistical analysis of the diameter of the inhibition zones.

After incubating for 24 h, the adherent bacteria on the 6 group samples were collected, diluted, and seeded on sterile agar plates to investigate the antibacterial rate by using the colony counting test (Fig. S2A to F). The SP-KZ and SP-OZ@KZ groups displayed the strongest antibacterial activities against all bacteria. Their antibacterial ratios all reached 90% at pH 5.0. The SP-Z and SP-OZ groups also showed a certain antibacterial ability, with the antibacterial ratios at pH 5.0 reaching about 30% against all bacteria. The bacterial live/dead staining results are consistent with colony counting tests (Fig. S2G to I). Figure 4D to I illustrate the result of the bacteriostatic ring test of each sample. As shown, no obvious bacteriostatic ring around PEEK and SPEEK was formed. In the case of the SP-Z and SP-OZ groups, an obvious bacteriostatic ring of approximately 2 to 3 mm was formed. In particular, the bacteriostatic rings increased to about 5 to 8 mm around the SP-KZ and SP-OZ@KZ groups, which showed a trend similar to that of the results mentioned above. In contrast, the SPEEK group did not demonstrate the ability to inhibit bacterial adhesion. The antibacterial properties of the samples were further characterized by SEM (Fig. 4A to C). *S. aureus*, *S. sanguis*, and *P. gingivalis* were on the PEEK and SPEEK surfaces with smooth membranes and had an intact regular shape and a relatively large density. The bacteria on SP-Z and SP-OZ surfaces showed a slight decrease in density compared to those on PEEK and SPEEK. Perforated and deformed membranes with a small amount of intact bacteria were observed on the SP-KZ and SP-OZ@KZ surfaces, which demonstrated the superior antibacterial capacity of SP-KZ and SP-OZ@KZ compared to that of other groups. KR12 could recruit negatively charged bacteria via the charge effect and disrupt the bacterial cell wall, which might lead to those fragmented membranes observed under SEM [32].

In this study, after loading KR12 onto the surface of SPEEK via ZIF-8 encapsulation, the modified material exhibited the highest antibacterial capacity among all samples, and no significant difference between the SP-KZ and SP-OZ@KZ groups was observed in the antibacterial experiments. The result verified the successful loading of KR12 within ZIF-8 encapsulation and indicated that KR12 could maintain its antibacterial capabilities when encapsulated within the ZIF-8 structure. The antibacterial capacity of all ZIF-8 loading groups increased as the pH decreased to 5.0, which confirmed the pH-responsive property and effectively abled control of possible bacterial infection during the implantation process. Nevertheless, the SP-Z and SP-OZ groups also possessed certain antibacterial capacities. This might be attributed to the degradation of ZIF-8 due to hydration–deprotonation and Zn^2+^ was then released. Zn^2+^ would interact with the thiol groups of bacterial respiratory enzymes, thereby inhibiting their function. The inactivation of respiratory enzymes leads to a disruption of the electron transfer process, resulting in the production of intracellular reactive oxygen species. Intracellular reactive oxygen species induce bacterial DNA damage, ultimately leading to bacterial death [33].

### In vitro cytotoxicity

**Fig. 5.**
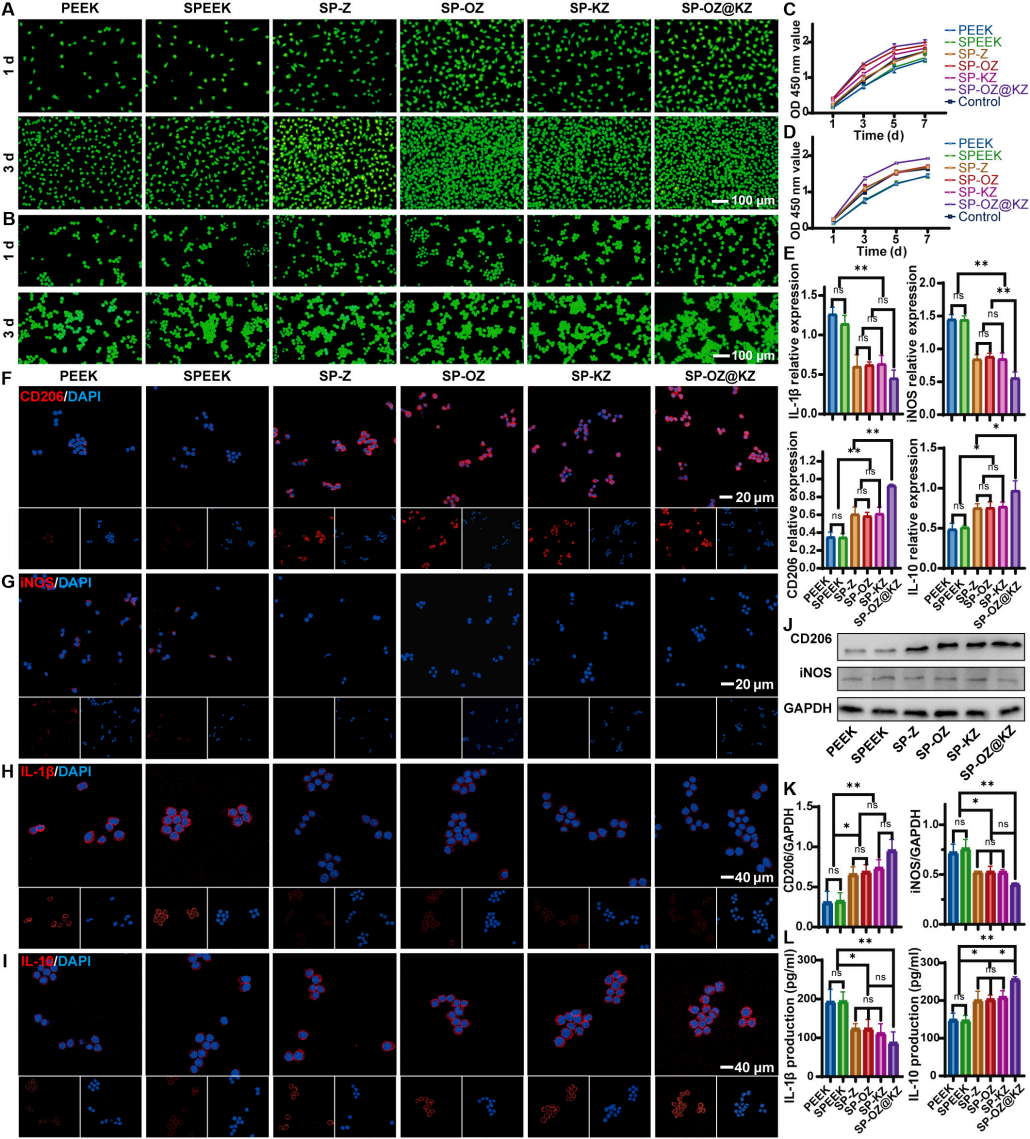
Biocompatibility of the samples and the effect of samples on the process of macrophage polarization. (A and B) Images of BMSC and RAW 264.7 cell live/dead staining after culturing for 1 and 3 d (green: live cells; red: dead cells). (C and D) Proliferation ability of BMSCs and RAW 264.7 cells by Cell Counting Kit-8 (CCK-8). (E) Real-time quantitative PCR (RT-qPCR) analysis of the expression levels of pro-inflammatory genes and anti-inflammatory genes related to macrophages. (F to I) Immunofluorescence analysis of CD206, iNOS, interleukin 1β (IL-1β), and interleukin 10 (IL-10) in RAW 264.7 cells (red: CD206, iNOS, IL-1β, and IL-10; blue: 4′,6-diamidino-2-phenylindole [DAPI]). (J and K) Western blot analysis of iNOS and CD206 and quantitative analysis of Western blot analysis. (L) IL-1β and IL-10 production analyzation by enzyme-linked immunosorbent assay (ELISA). OD, optical density; GAPDH; glyceraldehyde-3-phosphate dehydrogenase.

BMSCs and RAW 264.7 cells were seeded onto the samples to analyze in vitro biocompatibility in this study. The effect of the samples on cell proliferation was explored by CCK-8 assay (Fig. 5C and D). The proliferation of RAW 264.7 cells on SP-OZ@KZ was significantly increased than those of the other groups at 3 d, while there was no significant difference in the proliferation of BMSCs between the SP-OZ and SP-OZ@KZ groups at 3 d. However, with time prolongation, the proliferation ability of SP-OZ and SP-KZ increased, narrowing the gap with the SP-OZ@KZ group. The proliferation of BMSCs and RAW 264.7 cells on SP-Z, SP-OZ, SP-KZ, and SP-OZ@KZ reached the platform stage at 5 d. The results showed that the cell-proliferation-promoting ability of SP-OZ@KZ was significantly improved compared to that of PEEK, and all modified PEEK groups possessed relatively robust biocompatibility. Live/dead staining was also adopted to verify the biocompatibility of the samples (Fig. 5A and B). After culturing for 1 and 3 d, a high ratio of live cells was observed on all samples, and no significant dead cells were observed. The number of cells cultured with all samples also gradually increased over time. The result of the live/dead staining possessed a similar trend as the CCK-8 assay, indicating the samples to have high biocompatibility. The highest number of live cells with expanded polygonal shapes was observed in the SP-OZ@KZ group, while there was no significant difference between the SP-OZ and SP-OZ@KZ groups in co-culturing with BMSCs. The finding demonstrated that the SP-OZ@KZ group had the strongest effect in promoting cell proliferation. That might enable BMSCs to enter the osteogenic differentiation stage earlier, resulting in more new bone formation around the implants and robust bone–implant bonding in vivo [34].

### In vitro evaluation of macrophage polarization

As in other types of inflammation, macrophages in the peri-implant tissue of peri-implantitis patients tend to polarize to M1 macrophages. M1 macrophages serve a pro-inflammatory role induced by bacteria, releasing several pro-inflammatory substances such as iNOS, tumor necrosis factor alpha, IL-1β, IL-6, and IL-12. In addition, M1 macrophages activate osteoclasts and cause resorption of alveolar bone, while M2 macrophages serve an anti-inflammatory role, leading to tissue repair via several anti-inflammatory factors, such as CD206, IL-4, IL-10, IL-13, and transforming growth factor beta, and activate osteoblasts to promote bone regeneration. Immunomodulation of macrophages is essential for reliable osseointegration between the bone and the implant and for preventing peri-implantitis [35]. Therefore, the effect of the samples on the expression of inflammation-related factors in RAW 264.7 cells was investigated by immunofluorescence staining and qPCR analysis.

The results of immunofluorescence staining on iNOS and IL-1β (Fig. 5G and H) showed that the PEEK and SPEEK groups had the highest iNOS and IL-1β expression levels, and they were significantly decreased in the other groups. While the results of immunofluorescence staining on CD206 and IL-10 (Fig. 5F and I) showed the opposite trend, the expressions of CD206 and IL-10 were the highest in the SP-OZ@KZ group. The results revealed that the SP-OZ@KZ group could inhibit the polarization of macrophages to the M1 phenotype and activate polarization to the M2 phenotype significantly. The polarization of macrophages plays an important role in the regulation of the immune microenvironment. M1 and M2 macrophages play corresponding roles in different stages of bone healing. Excessive immune activation induced by bacteria leads to prolonged M1 polarization and chronic inflammation. Therefore, the inhibition of the polarization into M1 macrophages and the activation of the polarization into M2 macrophages were important for remodeling the local immune microenvironment and facilitating bone regeneration [2]. Similar results were also obtained with the qPCR analysis (Fig. 5E). The expression levels of pro-inflammatory genes released by the M1 macrophages (IL-1β and iNOS) were highest in the PEEK and SPEEK groups and decreased significantly in the other 4 groups. The expressions of anti-inflammatory genes released by the M2 macrophages (CD206 and IL-10) were highest in the SP-OZ@KZ group when compared with those in the PEEK and SPEEK groups. The protein expression levels of iNOS and CD206 in each group were further assessed by Western blot (Fig. 5J and K). The expression level of CD206 had a similar trend as the qPCR analysis. The expression of CD206 in SP-OZ@KZ was the highest among the groups, while those in the PEEK and SPEEK groups were significantly down-regulated relative to those in other groups. The expression levels of iNOS in the PEEK and SPEEK groups were higher than those in other groups. Nevertheless, there was no significant statistical difference between the SP-Z, SP-OZ, SP-KZ, and SP-OZ@KZ groups in the expression levels of iNOS. The levels of pro-inflammatory (IL-1β) and anti-inflammatory (IL-10) cytokines in the culture supernatants of each group were measured by ELISA kits (Fig. 5L). The pro-inflammatory cytokines in the SP-Z, SP-OZ, SP-KZ, and SP-OZ@KZ groups significantly decreased relative to those in the PEEK and SPEEK groups. Among them, there was a statistically insignificant decrease in SP-OZ@KZ relative to those in the other 3 groups. The level of IL-10 showed a trend opposite to that of IL-1β. SP-OZ@KZ had the highest level among all groups, while a significant decrease in IL-10 levels was observed in the PEEK and SPEEK groups.

In this study, all of the ZIF-8-modified PEEK groups could inhibit the polarization of macrophages to the M1 phenotype and activate the polarization to the M2 phenotype compared with the PEEK and SPEEK groups. The effects of the SP-OZ@KZ group were the strongest. The appearance of the above results should be due to the release of Zn^2+^ from the ZIF-8 coating on SPEEK. Several previous studies suggested that Zn^2+^ could modulate the macrophage phenotype from M1 to M2, as well as inhibit the pro-inflammatory responses and induce the anti-inflammatory responses [36]. Therefore, the introduction of Zn^2+^ on the SPEEK surface had a potentially positive effect on the modulation of the immune microenvironment and facilitation of bone regeneration. The relationship between Zn^2+^ and inflammatory signaling in macrophages relies primarily on TLR (Toll-like receptor) signaling (e.g., via TLR4), which is activated by the phosphorylation of interleukin-1 receptor-associated kinase 1 [37]. Zn^2+^ supplementation could down-regulate inflammatory cytokines (e.g., IL-1β and tumor necrosis factor alpha) through up-regulation of A20 to inhibit nuclear factor-κB (NF-κB) activation [38,39]. Therefore, the bold speculation was made that SP-OZ@KZ promoted macrophage M2-type polarization possibly by down-regulating the TLR4–NF-κB signaling pathway.

### In vitro osteogenic activity

**Fig. 6.**
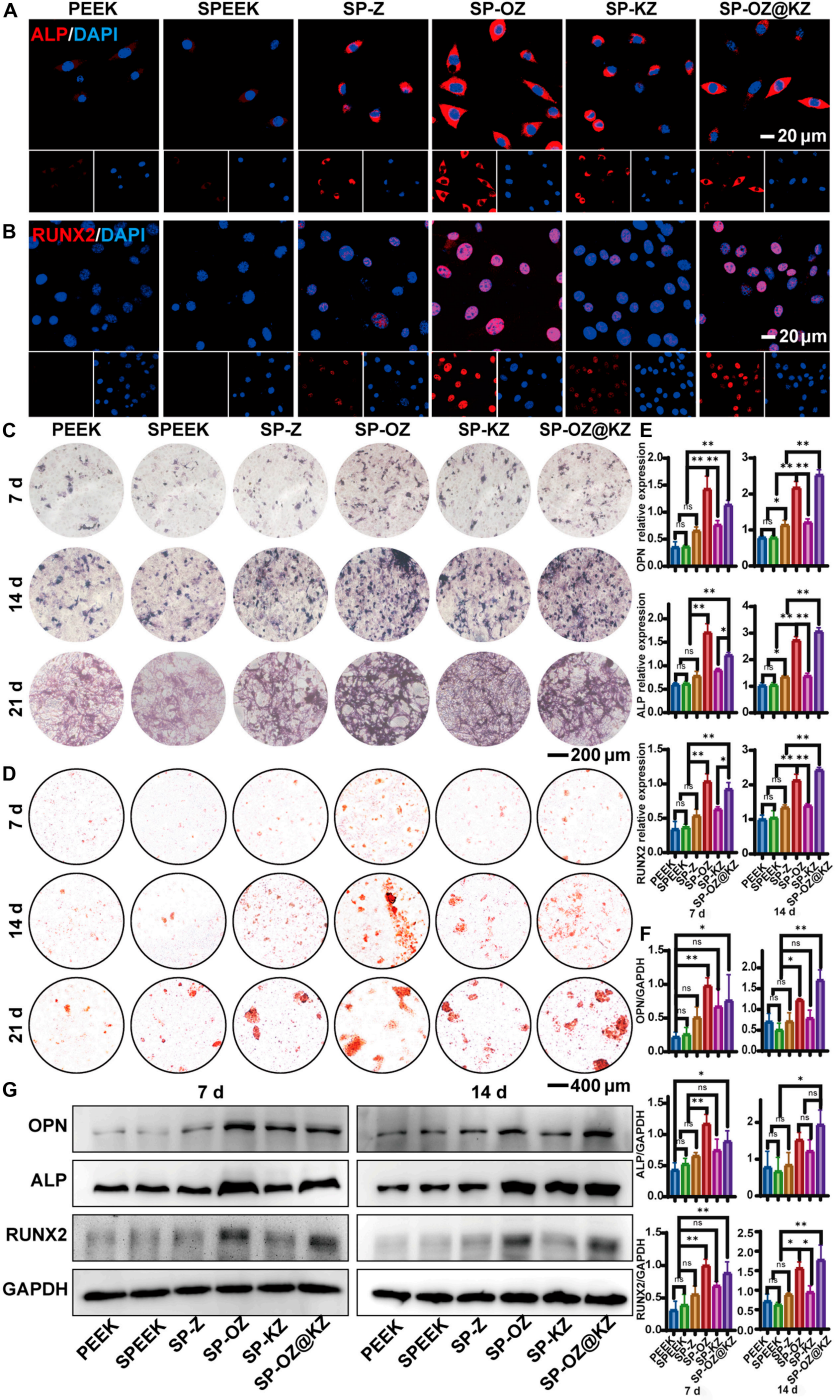
Effects of samples on osteogenic differentiation of BMSCs. (A and B) Immunofluorescence analysis of osteogenesis-related protein localizations in BMSCs (red: Runx2 and ALP protein; blue: DAPI). Microscopic images of ALP staining (C) and alizarin red staining assays (D). (E) RT-qPCR analysis of osteogenesis-related genes. (F and G) Western blot analysis of osteogenesis-related proteins and quantitative analysis of Western blot. One-way analysis of variance (ANOVA) was used for statistical analysis.

The new bone formation on the implant surface is a prerequisite for strong osseointegration. Therefore, the osteogenic capacities of the modified PEEK samples were evaluated in this study. ALP staining and ARS can reflect the degree of osteogenic differentiation. ALP is an early marker of osteoblast differentiation and maturation. ALP staining was adopted to detect the expression of ALP proteins in cells cultured with the samples visually [40]. The SP-OZ group showed the highest ALP activity among the samples, followed by the SP-OZ@KZ and SP-KZ groups after culturing for 7 and 14 d (Fig. 6C). After culturing for 21 d, the ALP activity of the SP-OZ@KZ group was increased to almost reach the level of that of the SP-OZ group. Similar results can be observed in ARS (Fig. 6D). ARS can stain the calcium nodules formed by calcium salt deposition, which is a crucial marker of the late stage of osteogenic differentiation. The calcium nodules increased and compacted over time in all groups. The highest number of calcium nodules was observed in the SP-OZ group at 14 d and changed to the SP-OZ@KZ group at 21 d. These results indicated that SP-OZ could enhance osteoblast differentiation early, while SP-OZ@KZ could enhance osteoblast differentiation at a late time, but the overall effect of SP-OZ@KZ was stronger than that of SP-OZ.

The effect of implants on cellular osteogenic differentiation is one of the key factors affecting bone regeneration. The functional role of osteoblasts in bone formation is divided into 3 main stages. The first stage is cellular adhesion and proliferation. The SP-OZ@KZ group was confirmed to promote the proliferation and adhesion of BMSCs in the above in vitro biocompatibility experiments. The second stage is the differentiation and maturation of osteogenic precursor cells into osteoblasts. The third stage is ECM mineralization, including calcium and phosphorus deposition [41]. In this study, *RUNX2*, *ALP*, and *OPN* were selected to evaluate the potential of the modified PEEK samples to promote osteogenic differentiation on BMSCs. Firstly, immunofluorescence staining was adopted to detect the localization of ALP and Runx2 in cells (Fig. 6A and B). The SP-OZ group and the SP-OZ@KZ group significantly increased the fluorescence intensity of ALP and Runx2, which indicated the SP-OZ and the SP-OZ@KZ coated on the SPEEK surface increased the osteogenesis-related protein deposition compared to that in the other groups significantly. Subsequently, the effect of samples on the expression of osteogenesis-related factors in BMSCs was assessed by Western blot (Fig. 6F and G) and qPCR assays (Fig. 6E). It was mentioned in the previous studies on osteogenesis-related factors that *RUNX2* is a major regulator that induces the differentiation of MSCs into mature osteoblasts [42]. *ALP* is considered to be related to phosphate formation, which is essential for pre-bone formation [43]. *OPN* is also confirmed to affect osteoblast migration, adhesion, and differentiation to form the bone matrix [44]. All of the osteogenesis-related proteins of the SP-OZ and the SP-OZ@KZ group were up-regulated significantly after culturing for 7 d compared to those of the other groups. The protein expression level of the SP-OZ group was the highest among all groups at 7 d, and that of the SP-OZ@KZ group was the highest at 14 d (*P* < 0.05). The changes in the gene expression levels of osteogenesis-related factors were investigated by qPCR assay. As shown in Fig. 6E, the mRNA levels of the above osteogenic factors of the SP-OZ and the SP-OZ@KZ groups were significantly higher than those of the other groups, and this trend was consistent with Western blot analysis. The SP-OZ and SP-OZ@KZ groups showed relatively strong osteogenic potential in this study, and both of them encapsulated OGPs. As previous studies reported, OGP could stimulate extracellular signal-regulated kinase 1 and 2 (ERK1/2) phosphorylation and lead to the enhancement of the expression of mitogen-activated protein kinase-activated protein kinase-2 [45]. OGPs could up-regulate *RUNX2* expression by mediating the ERK signaling pathway. RUNX2 further activates the expression of the important osteogenesis marker *ALP* [42]. Therefore, PEEK modified with OGP may produce more bone tissues around the implants through up-regulating osteogenesis-related genes by mediating the ERK signaling pathway, and more robust bone–implant integration is also expected in vivo.

### In vivo studies

#### In vivo anti-inflammation and Bacteriostasis assays

**Fig. 7.**
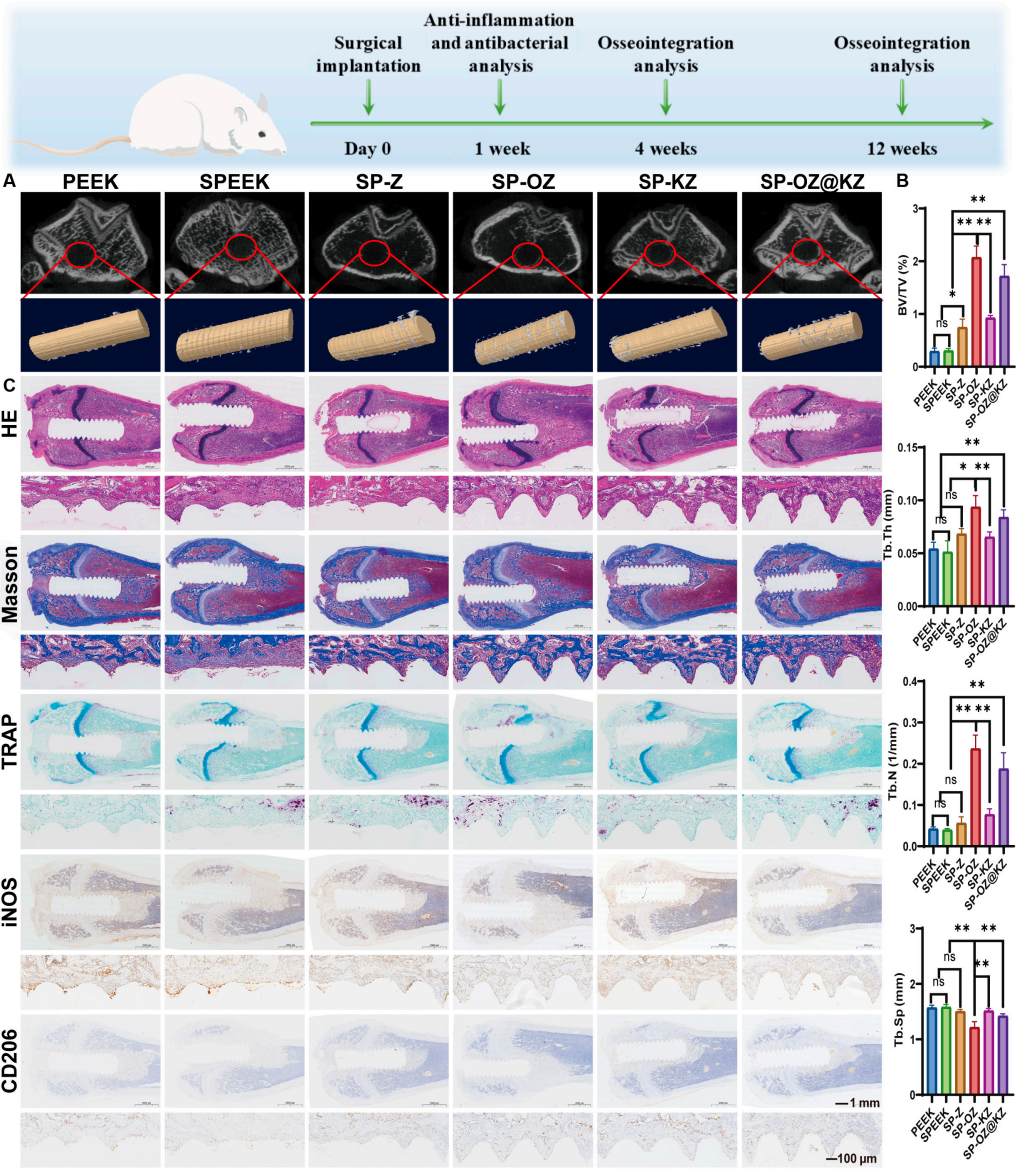
Osteogenesis and anti-inflammation effects of the samples after implantation for 1 week in the rats’ distal femur without infection model. (A) Three-dimensional (3D) reconstruction images of the rats’ distal femur without infection in different groups. (B) Bone volume over total volume (BV/TV), trabecular number (Tb.N), trabecular thickness (Tb.Th), and trabecular separation (Tb.Sp) of the distal femur without infection model. (C) Histological and immunohistochemistry (IHC) staining of the distal femur without infection model. HE, hematoxylin–eosin; TRAP, tartrate-resistant acid phosphatase.

The implants were implanted into rat femurs with or without bacterial infection to confirm the anti-inflammation and antibacterial effects of all samples. *S. aureus*, as a representative of the major bacterial components of orthopedic implant infections [46], was used to detect the in vivo antibacterial capacities of the implants in this study. During the in vivo study, the rats in all groups recovered well after surgery, with no significant systemic inflammation or death observed. The 3D reconstruction and the analysis of the micro-CT of the rat femur after implantation for 1 week revealed the SP-OZ group to have the highest bone volume surrounding the implants among all groups in the uninfected model (Fig. 7A and B). Nevertheless, there was no significant difference in the bone volume among all groups in the infected model (Fig. S3A and B). Large amounts of *S. aureus* were isolated from the PEEK and SPEEK groups, while that was significantly decreased in the SP-KZ and SP-OZ@KZ groups. The result of the colony counting test proved that the SP-KZ and SP-OZ@KZ implants exhibited significant antibacterial capacities (Fig. S4), consistent with the results of in vitro antibacterial experiments. Similarly, incomplete tissue morphologies with a large amount of fibrous tissues and neutrophil infiltration were observed in the HE- and Masson’s-trichrome-stained sections from the PEEK and SPEEK groups (Fig. 7C and Fig. S3C). The inflammation-related cytokine (iNOS and CD206) levels in the peri-implant tissues after implantation for 1 week were further investigated by IHC staining (Fig. 7C and Fig. S3C). The IHC staining revealed that the release of pro-inflammatory cytokine iNOS was significantly lower in the other 4 groups when compared with those in the PEEK and SPEEK groups. The lowest release among the 4 groups was observed in the SP-OZ@KZ group. The anti-inflammation cytokine CD206 was significantly highly released in the SP-OZ@KZ group. The results of IHC staining were consistent with those of HE staining; milder neutrophil infiltration was observed in the ZIF-8-modified SPEEK groups, which indicated an effective anti-inflammatory action of releasing Zn^2+^ from the ZIF-8 framework structure. Notably, the expression of iNOS in the SP-KZ group was also significantly lower than that in all other groups in the infection model. And the expression of CD206 was correspondingly higher. This suggests that the antimicrobial effect in vivo could effectively reduce the local inflammatory situation, which further led to higher osteogenesis in the SP-KZ group in the infection model. Thereafter, all of the implants’ effect on TRAP expression in peri-implant bone tissue was investigated (Fig. 7C and Fig. S3C). TRAP is recognized to be involved in osteoclast development, activation, and proliferation. The expression of TRAP is representative of osteoclast activity locally. TRAP-positive cells were observed in all groups. However, TRAP expression around the implant was significantly higher in the SP-OZ group, followed by the SP-OZ@KZ group in the uninfected model, while it was higher in the SP-KZ and SP-OZ@KZ groups in the infected model. As the precursors of the bone remodeling phase, the increase in osteoclasts might predict an earlier initiation of the osteogenesis phase [47]. Therefore, in order to further verify the longer-term effects of osseointegration after implantation, a series of in vivo experiments were performed after implantation for 4 and 12 weeks.

#### In vivo osteogenesis assays

**Fig. 8.**
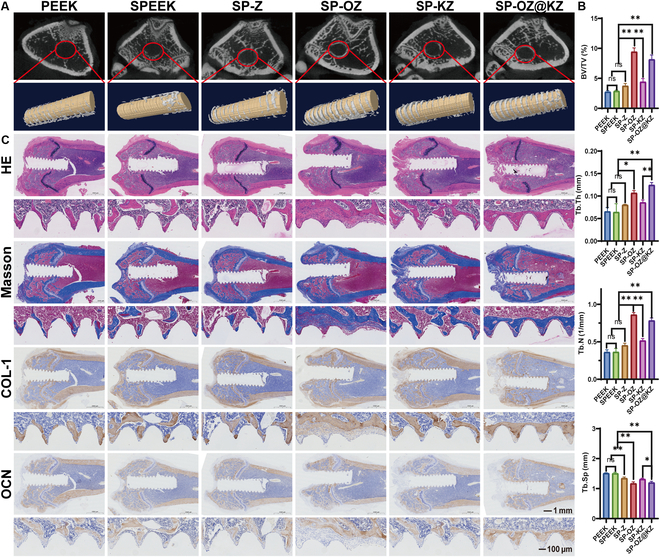
Osteogenesis effects of the samples after implantation for 4 weeks in the rats’ distal femur without infection model. (A) Three-dimensional reconstruction images of the rats’ distal femur without infection in different groups. (B) BV/TV, Tb.N, Tb.Th, and Tb.Sp of the distal femur without infection model. (C) Histological and IHC staining of the distal femur without infection model. COL-1, collagen-1; OCN, osteocalcin.

**Fig. 9.**
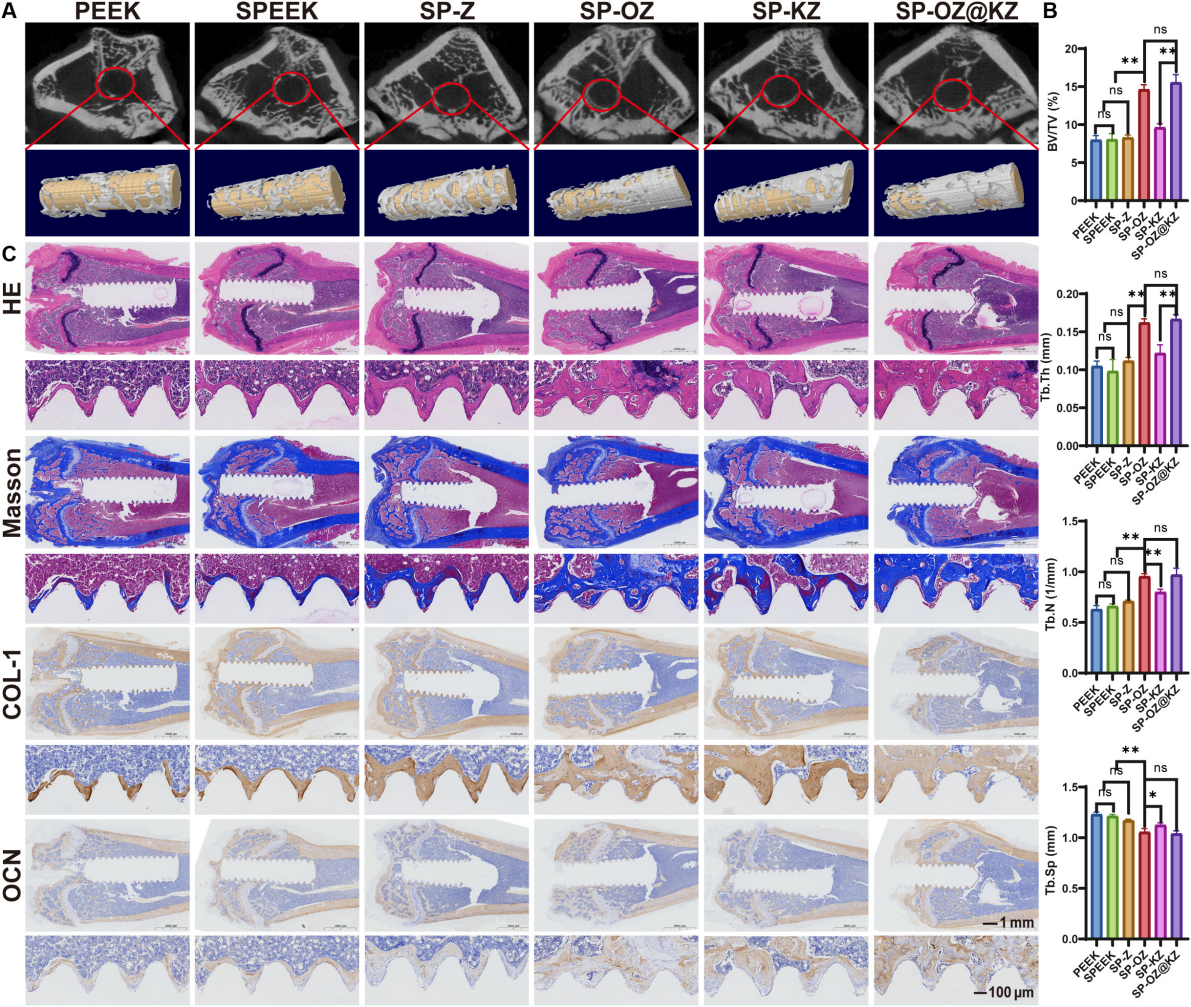
Osteogenesis effects of the samples after implantation for 12 weeks in the rats’ distal femur without infection model. (A) Three-dimensional reconstruction images of the rats’ distal femur without infection in different groups. (B) BV/TV, Tb.N, Tb.Th, and Tb.Sp of the distal femur without infection model. (C) Histological and IHC staining of the distal femur without infection model.

The osteogenesis efficiency of all implants was confirmed with the 3D reconstructed micro-CT images and their analysis after implantation for 4 and 12 weeks (Figs. 8 and 9 and Figs. S5 and S6). In the uninfected model at 4 weeks, a small amount of new bone was formed around the PEEK and SPEEK implants, whereas the highest amount of new bone was formed in the SP-OZ group, followed by the SP-OZ@KZ group. In contrast, after implantation for 12 weeks, the amount and maturity of the new bone formation in the SP-OZ@KZ group exceeded that in the SP-OZ group in the uninfected model. The amount of new bone formation in the infected model was generally less than that in the uninfected model in all groups after implantation for 4 and 12 weeks. Nevertheless, there was no significant difference in the amount of bone formation between the infected and uninfected models in the SP-KZ and SP-OZ@KZ groups. HE and Masson’s trichrome staining corroborated the above results (Figs. 8 and 9 and Figs. S5 and S6). The HE staining indicated pale pink osteoid around the implants in the uninfected model’s SP-OZ and SP-OZ@KZ groups. The result of Masson’s trichrome staining suggested that the peri-implant area of the other 4 groups was filled with blue-stained collagen connective tissues, indicating that the new bone tissue was not mature, whereas the area with new bone had more red stains in the SP-OZ and SP-OZ@KZ groups, suggesting that active mineralization occurred in the new bone tissue. However, in the infection model, the highest amount of new bone was observed in the SP-OZ@KZ group, which was significantly more than those of the other groups. The result might be attributed to the rapid release of KR12 due to the decreased pH in infected bone tissue. The rapid release of KR12 inhibited the extent of infection and, in conjunction with the subsequent release of OGP, promoted rapid peri-implant osteogenesis. The mineralization degree of new bone tissue formed in each group was observed by IHC staining (Figs. 8 and 9 and Figs. S5 and S6). OCN and COL-1 were selected for IHC staining. OCN is an important osteoblastic marker in the bone remodeling process, while COL-1, a marker of ECM formation, is considered critical for bone tissue mineralization [48]. The result of IHC staining showed that more OCN- and COL-1-positive cells were observed in the SP-OZ and SP-OZ@KZ groups than in the other groups in the uninfected model. Meanwhile, the OCN- and COL-1-positive areas were the largest in the SP-OZ@KZ group in the infected model. The results of IHC staining demonstrated that SP-OZ@KZ could not only promote the formation of new bone but also contribute to the mineralization of new bone to a certain extent. The effect was more prominent in infected bone tissue.

After implantation for 4 and 12 weeks, the SP-Z and SP-KZ groups showed osteogenic effects to some extent when compared to the PEEK and SPEEK groups. Previous studies demonstrated that Zn^2+^ participates in bone metabolism as a bone matrix component and an appropriate Zn^2+^ concentration could stimulate the proliferation and differentiation of osteoblasts [49]. KR12 was reported to activate the transcription of bone morphogenetic protein 2, which is one of the main factors inducing osteogenic differentiation of MSCs and endochondral ossification [50]. When SP-OZ@KZ implants were implanted in uninfected bone tissue, the bilayer ZIF-8 core–shell structure loaded on its surface degraded slowly due to the near-neutral intra-tissue pH. However, the osteogenic effects of Zn^2+^ and KR12 could compensate for the lack release of OGP to some extent in the early phase after implantation. The in vivo implantation experiments revealed that SP-OZ@KZ implants could inhibit the growth and proliferation of *S. aureus*. SP-OZ@KZ implants could rapidly promote the polarization of macrophages around the implant–bone interface toward the anti-inflammatory M2 phenotype and inhibit the progression of inflammation. SP-OZ@KZ implants could also inhibit local osteoclast activity in the early phase after implantation and effectively promote peri-implant osseointegration in the longer term after implantation. Its osseointegration effect is more prominent in infected bone tissues, as it can combine both antibacterial and anti-inflammatory effects at the same time and accelerate the release of antibacterial and osteogenic peptides in an acidic environment created by the decreased pH in infected bone tissues. Therefore, it might provide a new therapeutic idea for the prevention of the occurrence and progression of peri-implantitis.

## Conclusion

In summary, novel pH-responsive bilayer ZIF-8 core–shell structures with OGP wrapped in the inner core and KR12 wrapped in the outer shell were successfully encapsulated on the surface of SPEEK. The bilayer ZIF-8 core–shell coating could (a) disintegrate slowly and release Zn^2+^ and KR12 encapsulated in the outer shell at pH 7.4 to inhibit the adherence and growth of bacteria. It also delays the release of the OGP encapsulated in the inner core to coordinate the rate of decrease in implant stability. (b) When infection or inflammation occurs in the implant area, the pH decreases, and the release of Zn^2+^ and encapsulated KR12 and OGP accelerates, inhibiting infection and promoting rapid localized osteogenesis. The SP-OZ@KZ implants with good biocompatibility could also modulate macrophage M2 polarization to inhibit inflammation in in vitro and in vivo studies. Combining these features, as an overall consideration of the results of both infected and uninfected implantation models, the SP-OZ@KZ implants demonstrated optimal antibacterial, anti-inflammatory, and osseointegration effects in vivo. In conclusion, the SP-OZ@KZ implant is a promising peptide delivery implant system. The pH response properties are highly suitable for dental applications and provide a potential solution to the prevention of infections in the early stages of implantation.

## Data Availability

All necessary data are archived in the paper and Supplementary Materials and made available to any reader.
