# Supplementary material for: A pH-Responsive Polyetheretherketone Implant Modified with a Core–Shell Metal–Organic Framework to Promote Antibacterial and Osseointegration Abilities
Source: Biomater Res. 2025 Apr 25;29:0188. doi: 10.34133/bmr.0188 (PMC12022397; doi:10.34133/bmr.0188)
Supplement: Supplementary 1 — Figs. S1 to S6 Table S1 [file bmr.0188.f1.docx]

**A PH-responsive Polyetheretherketone Implant Modified With Core-shell Metal-Organic Framework to Promote Antibacterial and Osseointegration Abilities**

**Shiqing Ma^1,a,b^, Shiyu Yao^1,b,c^, Yumeng Li^1,b,c^, Yilin Yang^b,c^, Tianyi Tong^b,c^, Hong Zheng^b,c^, Beibei Ma^b,c^,** **Pengfei Wei^d^, Zhengyi Di^*,e^, Bo Zhao^*,d^, Jiayin Deng^*,b,c^**

^a^ Department of Stomatology, The Second Hospital of Tianjin Medical University, Tianjin 300070, PR China

^b^ Tianjin Key Laboratory of Oral Soft and Hard Tissues Restoration and Regeneration, No.12 Qixiangtai Road, Heping District, Tianjin 300070, PR China

^c^ Department of Periodontology, Tianjin Medical University School and Hospital of Stomatology, No.12 Qixiangtai Road, Heping District, Tianjin 300070, PR China

^d^ Beijing Biosis Healing Biological Technology Co., Ltd No. 6 Plant West, Valley No. 1 Bio-medicine Industry Park, Beijing 102600, PR China

^e^ College of Chemistry, Tianjin Key Laboratory of Structure and Performance for Functional Molecules, Tianjin Normal University Tianjin, 300387, PR China

Corresponding authors:

Zhengyi Di, E-mail: dzy@tjnu.edu.cn

Bo Zhao, E-mail: [zhaobo@biosishealing](mailto:liuzihao@tmu.edu.cn).com

Jiayin Deng, E-mail: [jdeng@tmu.edu.cn](mailto:jdeng@tmu.edu.cn)


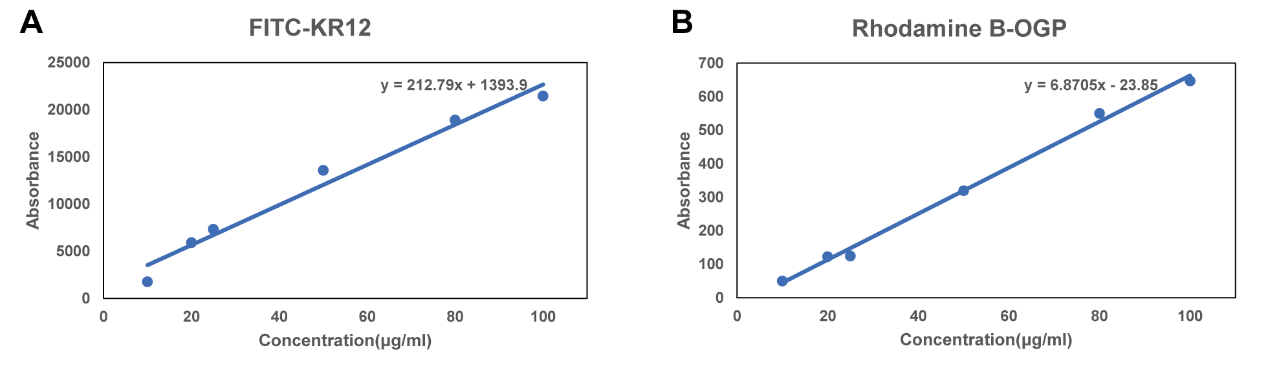


Fig. S1 Standard curves of (A) FITC-KR12 and (B) Rhodamine B-OGP peptides.

| Gene | Forward Primer (5’-3’) | Reverse Primer (5’-3’) |
| --- | --- | --- |
| ALP | CTGATCAGTGTGCCCCTGCAG | GGAGCTTGGAACGAATGTTCTG |
| RUNX2 | CCGAACTGGTCCGCACCGAC | CTTGAAGGCCACGGGCAGGG |
| OPN | AATGAAGGGCCCTGAGC | GCCAGTTCTGCAAGGAAGC |
| GAPDH | GACGGCCGCATCTTCTTGTGC | TGCAAATGGCAGCCCTGGTGA |
| IL-1β | TGGAGAGTGTGGATCCCAAG | GGTGCTGATGTACCAGTTGG |
| iNOS | CACCAAGCTGAACTTGAGCG | CGTGGCTTTGGGCTCCTC |
| CD206 | AGACGAAATCCCTGCTACTG | CACCCATTCGAAGGCATTC |
| IL-10 | GCTCTTACTGACTGGCATGAG | CGCAGCTCTAGGAGCATGTG |
| GAPDH | CTCCCACTCTTCCACCTTCG | TTGCTGTAGCCGTATTCATT |

Table S1. The primers of BMSCs and RAW264.7 cells were detected by qRT-PCR.


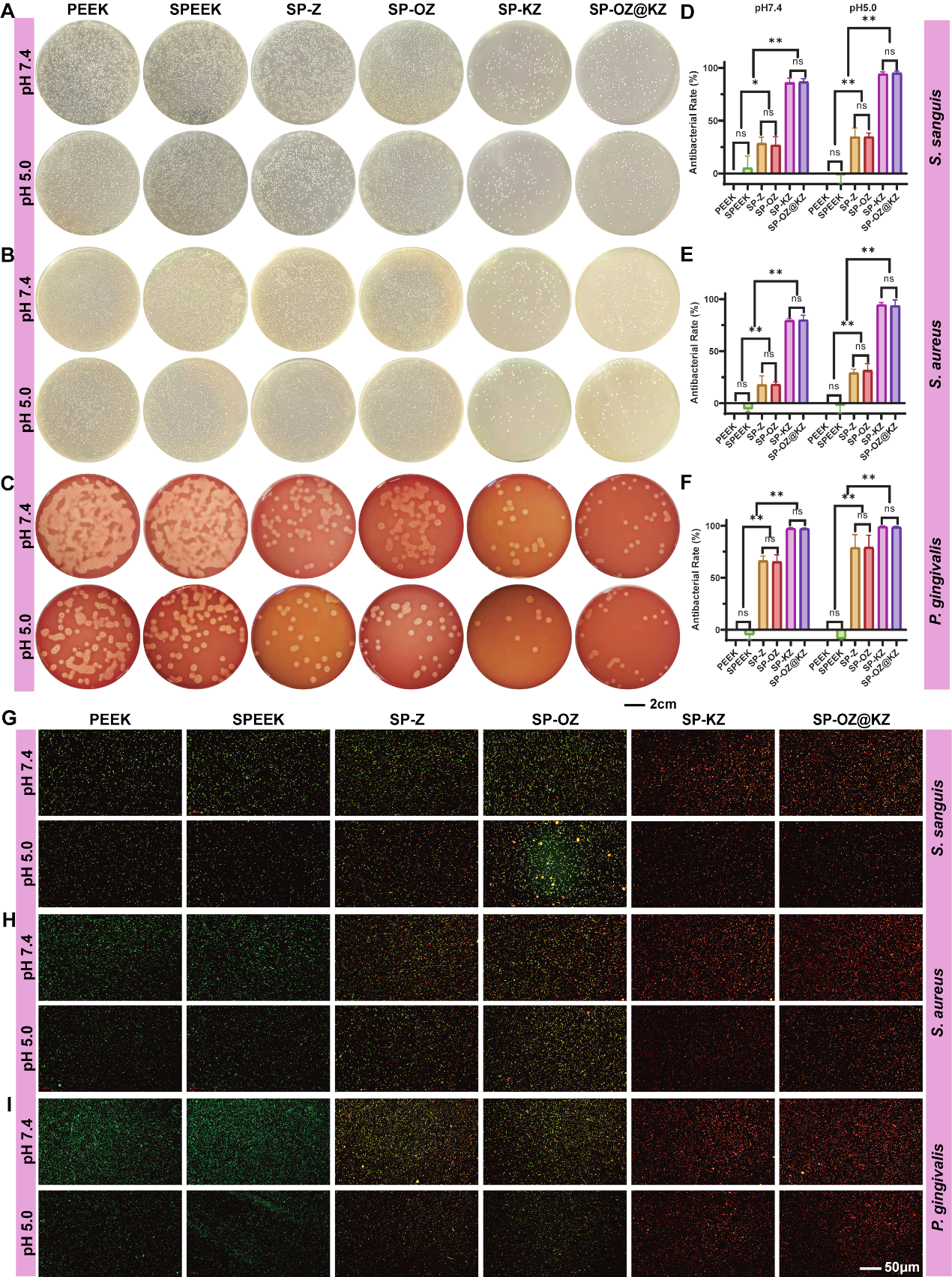


Fig. S2 Evaluation of antibacterial properties of samples. A-C) Bacteria colonies of S. aureus, S. sanguis, and P. gingivalis co-cultured with samples for 24 h. D-F) Statistical analysis of the antibacterial Rate. G-I) The live/dead staining of the bacteria on the surfaces of the samples visualized by fluorescence microscope.


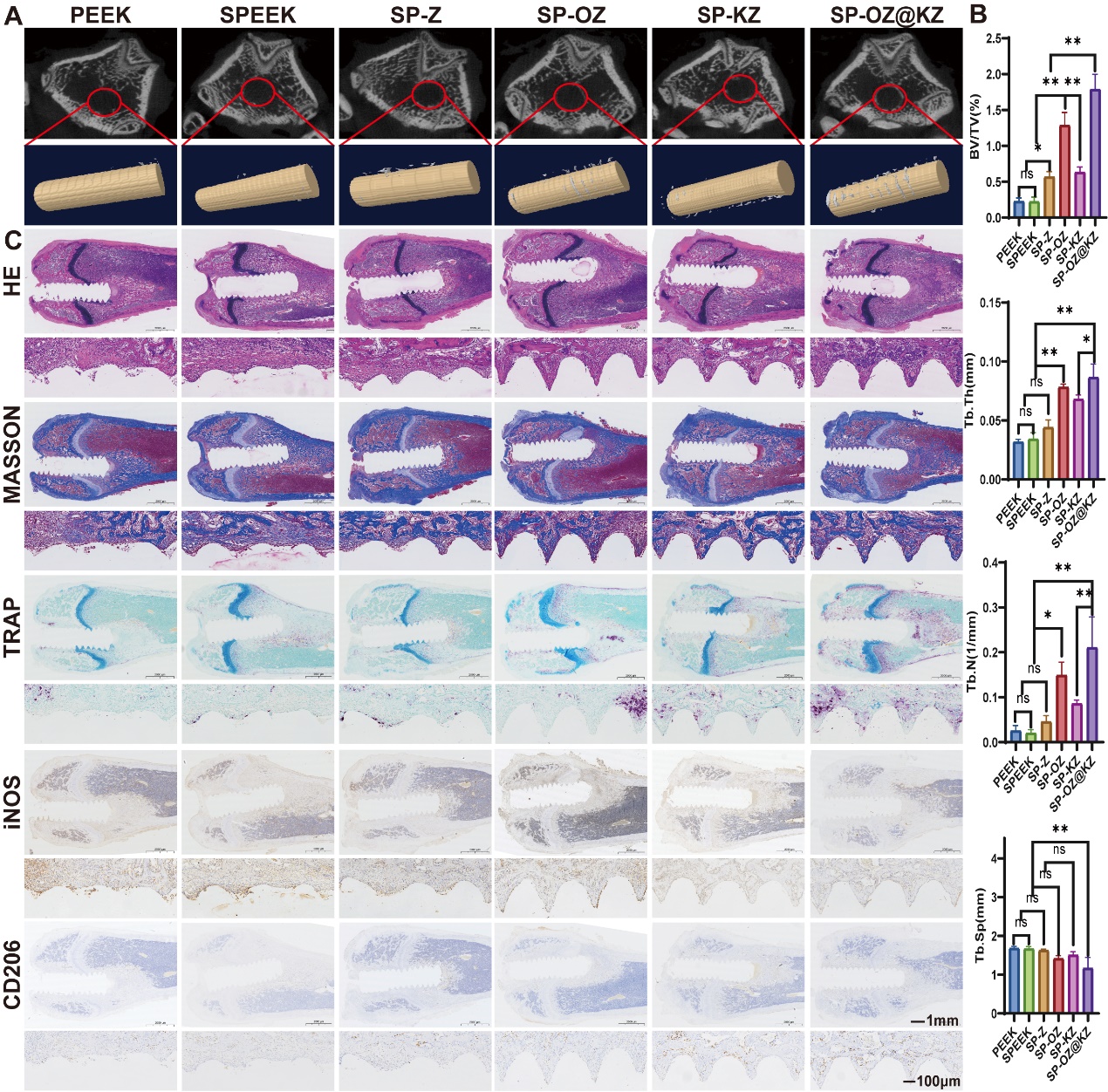


Fig. S3 Osteogenesis and anti-inflammation effects of the samples after implantation for 1 week in the rats’ distal femur with infection model. A) 3D reconstruction image of the rats’ distal femur with infection in different groups.. B) BV/TV, Tb.N, Tb.Th, and Tb.Sp of the distal femur with infection model. C) Histological and IHC staining of the distal femur with infection model.


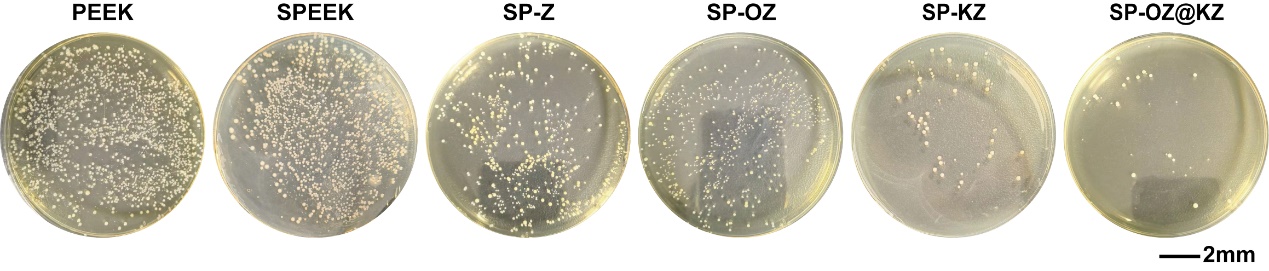


Fig. S4 The adherent bacteria colonies on the six group samples after implantation for 1 week in vivo.


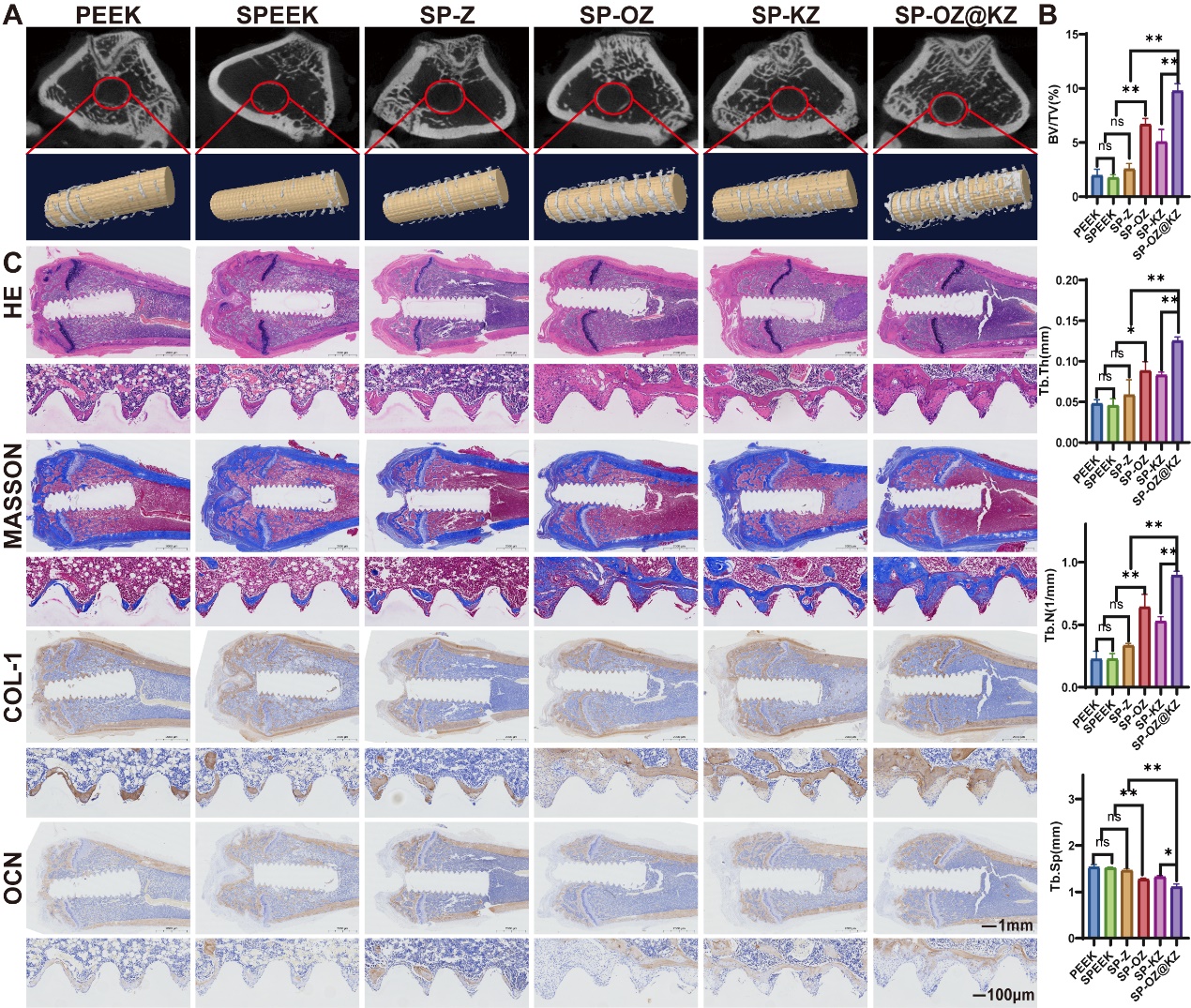


Fig. S5 Osteogenesis effects of the samples after implantation for 4 weeks in the rats’ distal femur with infection model. A) 3D reconstruction image of the rats’ distal femur with infection in different groups.. B) BV/TV, Tb.N, Tb.Th, and Tb.Sp of the distal femur with infection model. C) Histological and IHC staining of the distal femur with infection model.


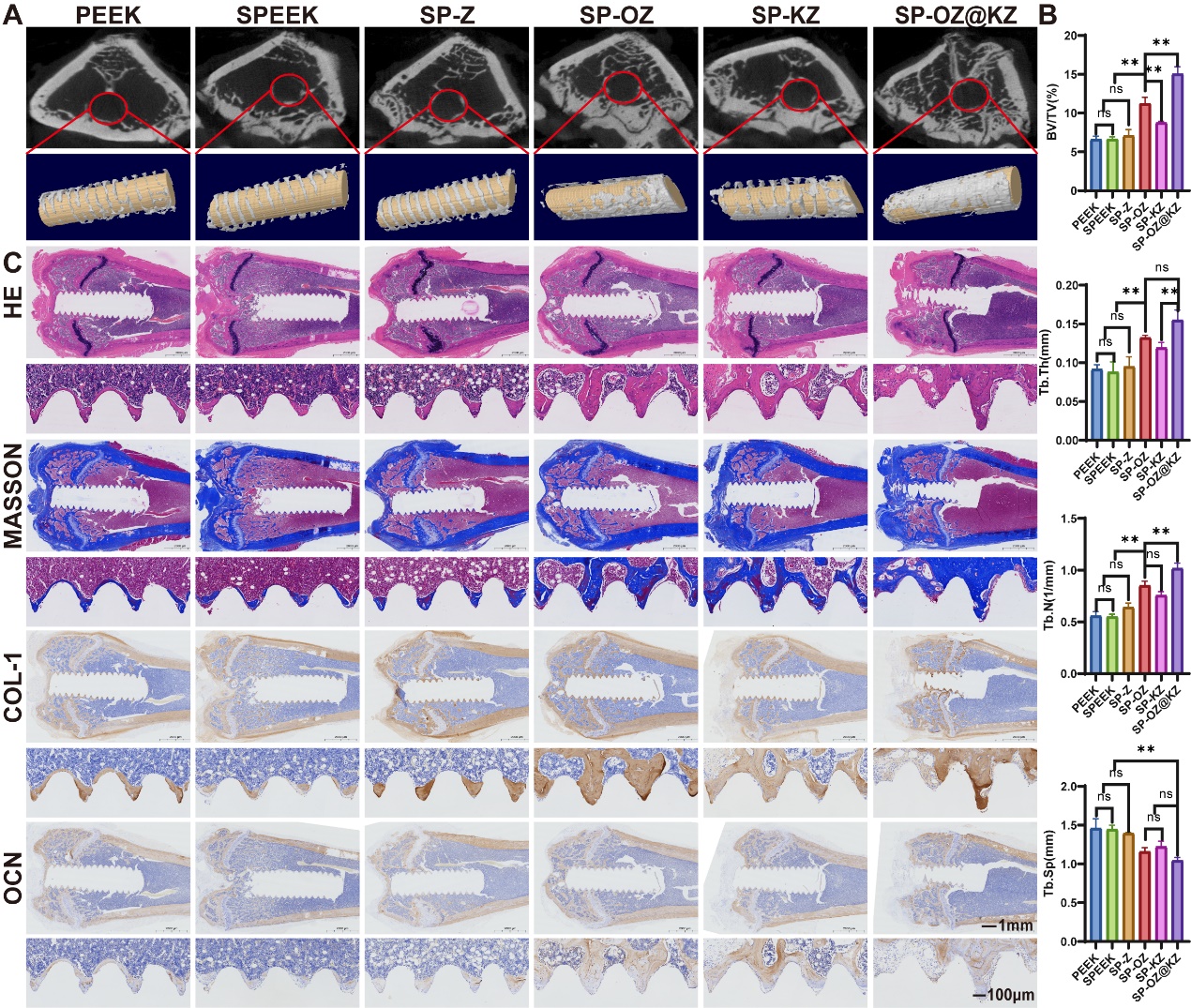


Fig. S6 Osteogenesis effects of the samples after implantation for 12 weeks in the rats’ distal femur with infection model. A) 3D reconstruction image of the rats’ distal femur with infection in different groups.. B) BV/TV, Tb.N, Tb.Th, and Tb.Sp of the distal femur with infection model. C) Histological and IHC staining of the distal femur with infection model.
